# REINFORCE-ING Chemical Language Models for Drug Discovery

**DOI:** 10.1021/acs.jcim.5c02053

**Published:** 2025-11-16

**Authors:** Morgan Thomas, Albert Bou, Jose Carlos Gómez-Tamayo, Gary Tresadern, Mazen Ahmad, Gianni De Fabritiis

**Affiliations:** † Computational Science Laboratory, Barcelona Biomedical Research Park (PRBB), 16770 Universitat Pompeu Fabra, C Dr. Aiguader 88, 08003 Barcelona, Spain; ‡ Department of Medical Sciences Khalifa University of Science and Technology, 127788 Abu Dhabi, UAE; § In Silico Discovery, Janssen Research & Development, 50148 Janssen Pharmaceutica N. V., Turnhoutseweg 30, B-2340 Beerse, Belgium; ∥ Institució Catalana de Recerca i Estudis Avançats (ICREA), Passeig Lluis Companys 23, 08010 Barcelona, Spain; ⊥ Acellera Labs, C Dr Trueta 183, 08005 Barcelona, Spain

## Abstract

Chemical language models, combined with reinforcement learning (RL), have shown significant promise to efficiently traverse large chemical spaces for drug discovery. However, the performance of various RL algorithms and their best practices for practical drug discovery are still unclear. Here, starting from the principles of the REINFORCE algorithm, we investigate the effect of different components from RL theory including experience replay, hill-climbing, baselines to reduce variance, and alternative reward shaping. We propose a new regularization method more aligned to REINFORCE than current standard practices, and demonstrate how RL hyperparameters can be fine-tuned for effectiveness and efficiency. Lastly, we apply our learnings to practical drug discovery by demonstrating enhanced learning efficiency on frontier binding affinity models by using Boltz2 as a reward model. We share our RL models used in the ACEGEN repository, and hope the experiments here act as a guide to researchers applying RL to chemical language models for drug discovery.

## Graphical abstract

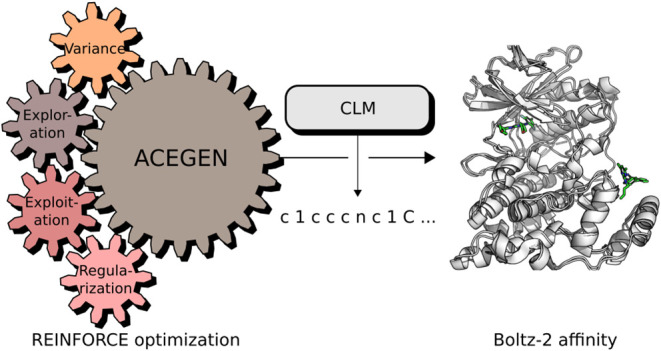

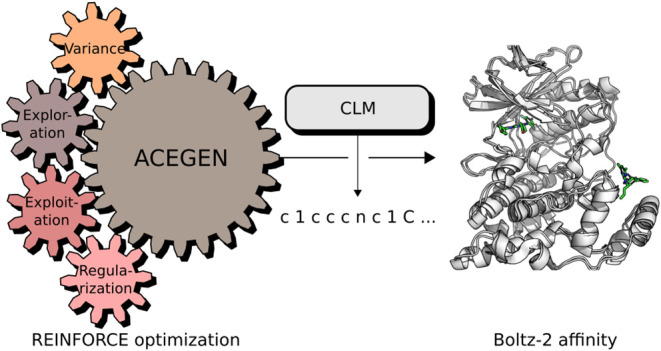

## Introduction

Chemical language models (CLMs)^1^ are a widely used^2^ and effective approach to de novo molecule generation.^3–5^ These models utilize molecular string representations to sequentially encode molecules one token at a time. In particular, the human-interpretable SMILES molecular grammar^6^ performs consistently well^7,8^ despite the advent of new machine-learning-inspired grammars.^6,9,10^ Furthermore, the sequential nature of token-by-token molecule generation can be framed as a decision-making problem in which reinforcement learning (RL) can be applied.

The combination of CLMs with RL^11^ is an evidenced strategy for a systematic exploration of chemical space.^12–14^ This approach leverages feedback from reward functions that assign a numerical value to molecular properties according to their desirability. Therefore, RL can progressively generate molecules that align better with predefined objectives, such as estimated potency, selectivity, bioavailability, or toxicity.

In recent work,^15^ we showed that REINFORCE-based^16^ algorithms remain the gold standard when considering different challenges associated with molecule generation for drug discovery. This also aligns with recent studies^17^ showing that when RL policies are pretrained, as in the case of large and chemical language models, REINFORCE can outperform other algorithms generally considered more advanced, such as Advantage Actor Critic (A2C)^18^ and proximal policy optimization (PPO).^19^ We also found that policy regularization via reward shaping or additional loss terms that encouraged the agent to remain close to a prior was more effective than the explicit addition of constraints to the reward functions for maintaining favorable chemistry in practice. Furthermore, we deconstructed REINVENT into its independent components: the REINFORCE algorithm, experience replay, reward shaping, and a likelihood regularization loss term. We discovered that while the reward shaping is effective for regularization, it is not easily interpretable, preventing fine-grained control of the trade-off between exploration and exploitation. Additionally, we found that the likelihood regularization term does not improve the performance of the REINFORCE algorithm.

In this paper, we test the effect of different orthogonal components inspired by RL literature on the REINFORCE algorithm. These include: the use of baselines to reduce variance in the gradient estimates, the impact of hill climbing by selecting the top-*k* elements in each data batch, different experience replay (ER) configurations, and a new more intuitive reward shaping that decouples the reward gradient from prior regularization. Lastly, we apply our learnings to a drug discovery challenge by optimizing frontier binding affinity models^20^ to identify putative allosteric JNK3 ligands.

## Methods

### Chemical Language Models Combined with Reinforcement Learning

CLMs^1^ that utilize language representation of chemistry such as SMILES^6^ or DeepSMILES^9^ have shown promising success in de novo drug discovery.^12,21–23^ Typical autoregressive models are trained unsupervised on a corpus of molecules at the task of “next token prediction”, where all tokens combined sequentially form the full language representation of a molecule.^3^ A trained model can sample new tokens conditional upon previous tokens, such that the resulting molecules are likely to adhere to chemical rules and belong to a similar chemical distribution as the training molecules.^3,5^

From an RL perspective, the problem of sequentially building molecules using tokens can be viewed as navigating a partially observable Markov decision process (MDP),^11^ described by the quintuple ⟨*S*, *A*, *R*, *P*, ρ_0_⟩. Here, *S* represents the set of all possible states in the problem space, and *A* denotes the set of valid actions available to the agent. The reward function, R:S×A×S→R , assigns numerical values to transitions from one state to another based on the action taken. The transition probability function, P:S×A→P(S) , specifies *P*(*s*_*t*+1_|*s*, *a*) as the probability of transitioning to state *s*_*t*+1_ from the current state *s* under action *a*. Lastly, ρ_0_ signifies the initial state distribution. In this context, a parametrized RL policy (a pretrained CLM), denoted as π_θ_, guides molecule building by successively processing the current state, which represents a partially built molecule, and selecting actions *a*_*t*_ that represent the next token at each step, until reaching a terminal state. The reward function evaluates the molecule with respect to a defined objective/s, assigning a scalar value either at each generative step or at the end of the process when a complete compound is produced. Within this framework, π_θ_(*a*_*t*_|*s*_*t*_) denotes the probability of the policy function, parametrized by θ, taking action *a*_*t*_ in state *s*_*t*_. The sequence τ represents a complete series of actions needed to construct a molecule, often referred to as an episode. *P*(τ|θ) indicates the probability of a trajectory τ given the policy parameters θ. Additionally, *R*(τ) represents the cumulative sum of rewards over the trajectory τ.

### Reinforcement Learning with REINFORCE

REINFORCE^16^ is a policy gradient RL algorithm that seeks to learn policy parameters θ based on the gradient of a scalar performance measure *J*(θ), aiming to maximize performance as shown in eq 1. Compared to algorithms like proximal policy optimization (PPO), which are generally considered more advanced, REINFORCE offers lower computational costs and is less sensitive to hyperparameter tuning. PPO emphasizes stability through small, stable updates and is suited for scenarios with large off-policy gradient updates, a regime that dominates traditional deep RL benchmarks. However, this is not necessarily the case with pretrained CLMs, where the policy weight initialization is not random due to unsupervised pretraining on a corpus of molecules constituting a prior policy. Additionally, in our setting, rewards are exclusively assigned to full generations, lacking true rewards for intermediary actions (or tokens in CLMs). Both of these characteristics align better with REINFORCE, which allows larger gradient updates and treats the entire generation as a single action. This hypothesis was validated in the context of RL from human feedback (RLHF),^17^ and contributes to understanding why state-of-the-art methods for language-based drug discovery are based on REINFORCE.

1 $$\nabla J\left(\theta \right)=E_{\tau ∼\pi_{\theta }}\left[\sum_{t=0}^{T}\nabla_{\theta }\;\log\;\pi_{\theta }\left(a_{t}|s_{t}\right)\cdot R\left(\tau \right)\right]$$

### Extensions to REINFORCE

By itself, REINFORCE has demonstrated effectiveness for language based drug discovery, as shown in our previous work.^15^ However, to further enhance its performance for the problem at hand, several orthogonal extensions of this algorithm have been applied in the literature to better optimize the reward function and generate chemically diverse, property-optimized molecules.^12,14,17,24–27^ We re-evaluate a selection of extensions, aiming to combine them to create an integrated agent.

#### Baselines

To enhance learning, the variance of the estimator in eq 1 can be reduced by subtracting any baseline *b* as in eq 2 which only depends on the state.^11^ We consider two options: a moving-average baseline (MAB) and a leave-one-out baseline (LOO).^17^

2 $$\nabla J\left(\theta \right)=E_{\tau ∼\pi_{\theta }}\left[\sum_{t=0}^{T}\nabla_{\theta }\;\log\pi_{\theta }\left(a_{t}|s_{t}\right)\cdot \left(R\left(\tau \right)-b\right)\right]$$

#### Hill-Climb

Hill-Climb (HC) is a strategy where only a specific ratio of molecules generated by the agent is retained at each iteration and used on the training process. These selected molecules are chosen based on their reward ranking, with only the top-*k* performing molecules being kept. This method has been demonstrated to enhance learning efficiency in language-based de novo molecule generation.^14^ In our study, we delve further into this approach by investigating the effects of retaining various portions of the data batch.

#### Experience Replay

While REINFORCE relies solely on on-policy data to refine its policy, some works have explored augmenting its learning process with off-policy data. This strategy involves storing the molecules with the higher rewards encountered during training, then randomly sampling batches of these off-policy data points. These batches are subsequently combined with the on-policy data batches generated by the agent during training. This approach has demonstrated enhanced sample efficiency in algorithms such as REINVENT^24^ and PPOD.^28^ Notably, previous works primarily focused on sampling off-policy molecules with priority proportional to their reward. However, our study also explores a uniform sampling strategy.

#### Policy Regularization via Reward Shaping

Reward shaping is the process of augmenting the rewards of RL agents to guide them toward desired behaviors that might not be explicitly captured by the original reward.

The REINVENT algorithm^12,25^ proposes a reward shaping formulation that couples the agent policy to the prior policy to regularize agent learning of an arbitrary reward function while maintaining important aspects of the prior policy learned, in this case, valid chemistry that resides in a similar chemical space to the prior unsupervised training data set. This formulation is shown in eq 3, where π_prior_ is the log-likelihood of generating a specific molecule by the prior policy, π_agent_ is the log-likelihood of generating a specific molecule by the RL agent, and σ is a hyperparameter.

3 $$R{\left(\tau \right)}_{reshaped}=\frac{{\left(\pi_{prior}-\pi_{agent}+\sigma \cdot R\left(\tau \right)\right)}^{2}}{\pi_{agent}}$$

Here we propose to simplify the REINVENT formulation of REINFORCE while maintaining regularization to the prior policy π_prior_. First, we recognize that REINVENT seeks to minimize the difference between the prior and agent policies even if the prior likelihood is low, we alter this behavior such that high prior likelihood is favorable, and low prior likelihood is unfavorable by simply adding the prior policy. Second, we decouple the hyperparameter σ and assign it to control the effect size of regularization to the prior policy and then we add an exponential coefficient α to investigate effect of increasing the gradient of the reward landscape *R*(τ). This is shown in eq 4, note the clip term to ensure the reshaped reward is never negative.

4 $$R{\left(\tau \right)}_{reshaped}=clip{\left(R\left(\tau \right)+\sigma \cdot \pi_{prior}\right)}^{\alpha }$$

#### Policy Regularization via Kullback–Leibler Divergence

Alternatively, an additional Kullback–Leibler (KL) divergence loss term can be used to regularize the agent policy (see eq 5). This is a measure of the difference in the distribution of action probabilities given a state between two policies.

5 $$\begin{array}{l} KL\left(\pi_{prior}∥\pi_{agent}\right)=\\ \lambda_{KL}\cdot \sum_{t=0}^{T}\sum_{a_{i}\in A}\pi_{prior}\left(a_{i}|s_{t}\right)\;\log\left(\frac{\pi_{prior}\left(a_{i}|s_{t}\right)}{\pi_{agent}\left(a_{i}|s_{t}\right)}\right) \end{array}$$

#### Exploration via Reward Shaping

The use of diversity filters to penalize molecules already generated can also be considered a form of reward shaping. This could be penalizing repeated episodes (or in our context molecules), or penalization of repeated sampling in areas of chemical space recorded by clustering previously generated molecules into different bins via similarity measures.^27,29^ This is due to the dynamic modification of the reward depending on the output of the diversity filter that changes during optimization. This type of reward shaping can be used to penalize excessive exploitation and hence, encourage exploration into different solution spaces.

#### Exploration via Additional Loss Terms

One common method to prevent overexploitation and to encourage exploration is an entropy (ENT) penalty term as shown in eq 6. Less common is a high agent log-likelihood (ALL) penalty term to penalize very likely sequences. This is implemented in the original REINVENT algorithm and shown in eq 7.

6 $$ENT=-\lambda_{ENT}\cdot \sum_{t=0}^{T}\sum_{a_{i}\in A}\pi_{\theta }\left(a_{i}|s_{t}\right)\;\log\pi_{\theta }\left(a_{i}|s_{t}\right)$$

7 $$\mathrm{ALL}=-\lambda_{\mathrm{ALL}}\cdot \sum_{t=0}^{T}\log\pi_{\theta }{\left(a_{t}|s_{t}\right)}^{-1}$$

#### Exploration via Random Network Distillation (RND)

RND^26^ utilizes the error between a target *f* and estimator neural network f̂ to provide a low-overhead intrinsic reward bonus that encourages exploration. The estimator network is trained to estimate the maximum squared error between network outputs given an input ${∥\mathrm{f̂}\left(x;\theta \right)-f\left(x\right)∥}^{2}$ . This leverages the epistemic uncertainty of neural networks depending on the quantity of training data, i.e., few training examples leads to a large error and therefore a large exploration bonus and vice versa (eq 8). This approach is low overhead because it does not require a memory lookup of previously generated examples as required by diversity filters. This has already proved effective at increasing diversity in CLMs trained with RL.^27^

8 $$R\left(\tau \right)=R\left(\tau \right)+\lambda_{RND}\cdot {∥\mathrm{f̂}\left(x;\theta \right)-f\left(x\right)∥}^{2}$$

### Model

In this work, we utilize a recurrent neural network with gated-recurrent units as the policy model, pretrained on a subset of molecules extracted from ChEMBL28 ^30^ as described previously.^15^ All algorithms were implemented or reimplemented in ACEGEN for consistency.

### MolOpt Benchmark and Performance Metrics

The MolOpt benchmark^31^ was used as implemented in MolScore^32^ with a budget of 10,000 molecules and optimization was repeated with 5 replicates. This benchmark describes 23 distinct tasks, each associated with different targets. To measure performance, we focus on eight metrics summed over all 23 objectives as described in our previous work^15^ (a perfect score for all metrics is 23). The first four metrics are “chemistry-naive” proxies for validity, effectiveness (to what extent can the objective score be optimized), efficiency and exploration. The second four are “chemistry-aware” equivalents for the same.

At the first “chemistry-naive” level we treat the objective as a perfect description, where the reward provided covers all aspects of chemical desirability.

#### Valid

Validity measured by the proportion of chemically correct molecules.

#### Top-10 Avg

Effectiveness measured by the average reward of the best 10 molecules.

#### Top-10 AUC

Efficiency measured by the area under the curve of the best 10 molecules cumulatively throughout optimization.

#### Unique

Exploration measured by the number of unique molecules generated throughout optimization.

At the second “chemistry-aware” level we assume an imperfectly described objective that reflects a more realistic perspective for drug discovery. Where B&T-CF refers to “basic” chemistry filters and includes a set of substructure alerts, Log *P* range, molecular weight range, number of rotatable bonds and where “target” chemistry filters include Log *P* and molecular weight range relative to a target data set (here the pretraining data set from ChEMBL28), as well as an ECFP-based outlier analysis. For specific details see Bou et al.^15^

#### B&T-CF

Validity measured by the proportion of molecules not considered to have highly idiosyncratic atomic environments or property space relative to the pretraining data set.

#### B&T-CF Top-10 Avg (Div)

Effectiveness measured by the Top-10 Avg after applying B&T-CF filters and enforcing that the best 10 molecules represent a diverse selection of chemotypes.

#### B&T-CF Top-10 AUC (Div)

Efficiency measured by the Top-10 AUC after applying B&T-CF filters and enforcing that the best 10 molecules represent a diverse selection of chemotypes.

#### B&T-CF Diversity (SEDiv@1k)

Exploration measured by the sphere exclusion diversity metric^33^ of generated molecules after applying B&T-CF filters. This measures the chemical space coverage of the sample size, here 1000. A value of 1 can be interpreted as every molecule covering a “different part” of chemical space, while close to 0 means that all molecules cover the same part of chemical space.

## Results and Discussion

### Exploitation-Regularization Trade-Off

Maintaining the learnings of a prior policy during RL can help to regulate chemistry to be similar to the prior training data set which results in the generation of chemistry in desirable chemical spaces. While the reward shaping used by REINVENT helps in practice to regularize learning to the prior policy, the specific formulation we found to be nonintuitive. It is challenging to understand the resulting reward landscape and how to obtain fine-grained control over the trade-off between optimization and regularization. In addition, the numerator is arbitrarily scaled to the power of 2 leading to a steeper gradient in the reward landscape. To better understand the reward landscape from a REINFORCE perspective, we visualized the reward landscape in Section SA. The reward landscape (Figure S1) indicates several curious behaviors. First, in some situations for lower σ values, there exists a region of high reshaped reward for very low prior likelihoods and low reward which is actually undesirable. We note that although this idiosyncratic region exists, it may never be encountered in practice at such prior low likelihood values. Second, the σ value not only affects the shape of the landscape but also the scale and gradient of the reshaped reward leading to one hyperparameter controlling two effects during reshaping. Therefore, we sought an alternative formulation for reward shaping to regularize the REINFORCE algorithm to the prior policy. The outcome of our simplified reshaped reward landscape is visualized in Figure S2, where it can be seen that the shape of the reshaped reward landscape changes with respect to the prior likelihood depending on σ, the gradient changes depending on α acting independently, while the scale of reshaped reward remains consistent between 0 and 1.

We tested this new reward-shaping mechanism to balance the trade-off between optimization and regularization to a prior policy when conducting RL with REINFORCE. To investigate the behavior of our proposed reward shaping, we titrated the two hyperparameters α and σ and conducted RL on an example objective from the MolOpt benchmark, maximization of JNK3 estimated probability of bioactivity. This task is prone to “hacking” where the generative model can drift into undesirable property spaces and generate impractical chemistry.^34^ The results are shown in Figure 1. First, σ successfully regularizes RL with increasing values resulting in lower prior negative log-likelihoods (NLL) during training and thus molecules are more likely sampled from the prior policy. Moreover, this remains true at all values of α. Meanwhile, increasing values of α successfully results in more efficient optimization, with score optimization shifting from linear to sigmoidal in nature over the budget of 10,000 molecules. Lastly, it can be seen that higher values of σ appear to slightly shift the learning curve of the JNK3 score to the right, i.e., learning takes slightly longer. The additional constraint to the prior policy effectively adds a potentially inhibitory learning objective. Overall, the reward shaping demonstrates independent and successful control over both return gradient and prior policy regularization not possible with popular alternatives like REINVENT (see Figure S3).

**Figure 1:**
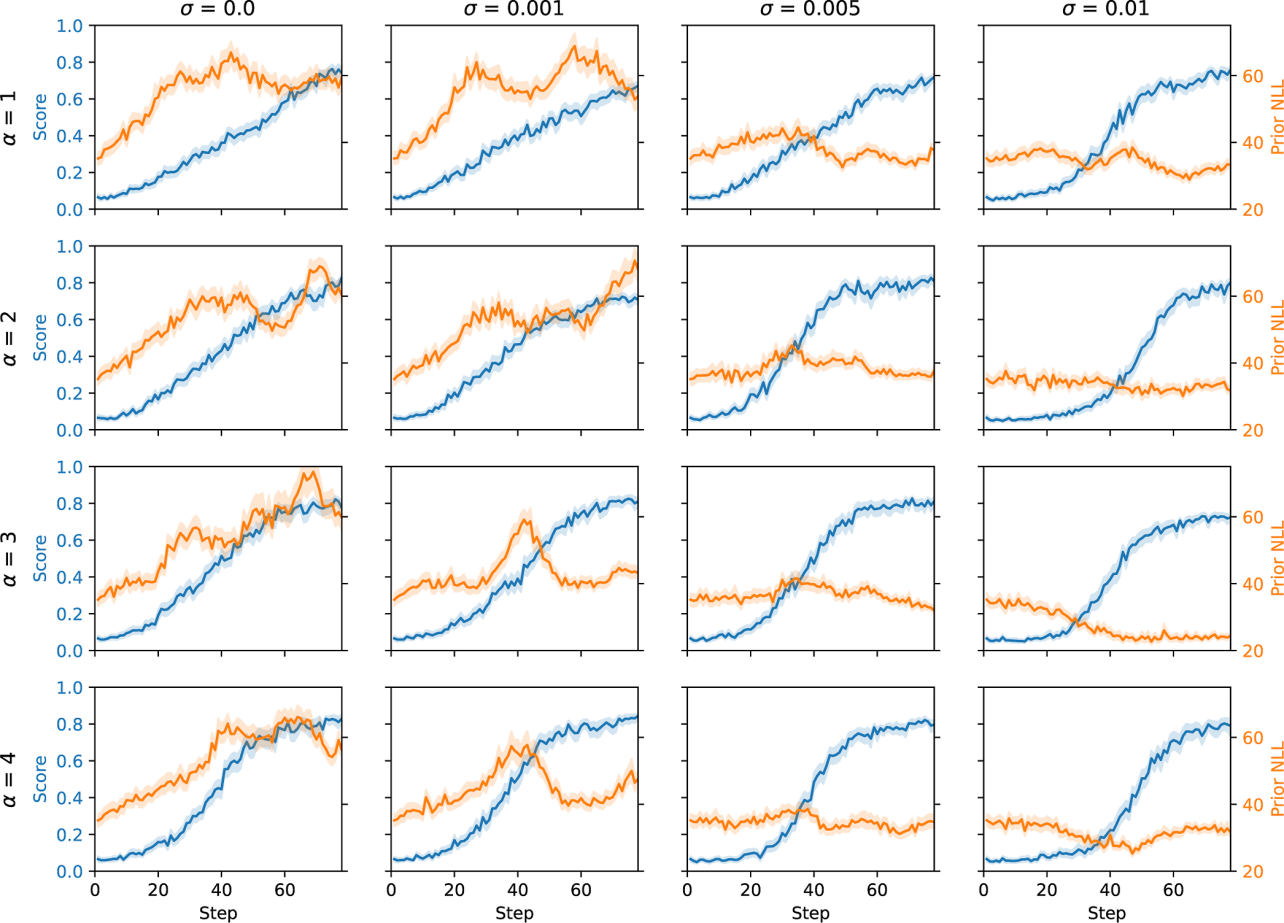
Performance of REINFORCE with the proposed reward shaping at different values of α and σ on the JNK3MolOpt benchmark task. On the left *y*-axis (blue) is the JNK3 score during the training step, while on the right *y*-axis (orange) is the negative log-likelihood (NLL) (lower is more likely, and hence better) of molecules according to the prior policy. Variables are measured during RL training steps until 10,000 molecules have been evaluated. Note that higher α increases learning efficiency and higher σ increases the prior likelihood, giving control over exploitation-regularization trade-off.

### Extending REINFORCE

We also evaluated the performance of each REINFORCE extension independently with respect to plain REINFORCE on the MolOpt benchmark to better understand the benefits of each.

#### REINFORCE Baselines

To reduce the variation in the estimated policy gradients, we investigated the subtraction of a baseline using two methods (1) subtraction of a moving average baseline and (2) subtraction of a leave-one-out baseline. Figure 2 shows that the subtraction of either of these baselines improves the effectiveness by ∼6% and efficiency by ∼4%. This comes at a slight compromise with a drop in the number of unique molecules of ∼3%, indicating less exploration which is likely a factor of enhanced optimization. Overall the moving average baseline appears to show the best overall profile for performance improvement over REINFORCE.

**Figure 2:**
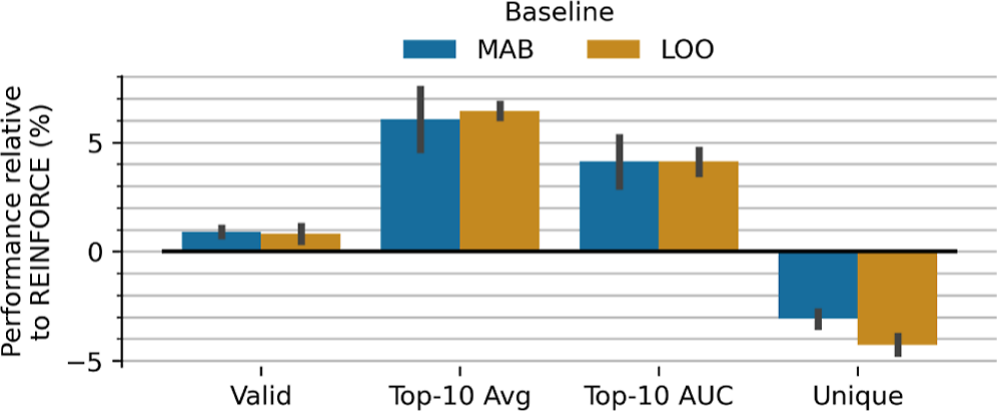
Effect of moving average baseline (MAB) and a leave-one-out baseline (LOO) compared to REINFORCE without baselines. Both baselines increase validity and effectiveness at a small cost to exploration.

#### Hill-Climb

Hill-climb is a strategy that subsets the highest scoring molecules on-policy; we evaluate different top-*k* values and their effect on learning efficiency shown in Figure 3. We observe that as the top-*k* ratio decreases, the effectiveness and efficiency increase correspondingly. This strategy can considerably boost effectiveness as much as ∼10% and efficiency as much as ∼6% with only small drops in validity and uniqueness of ∼3% and ∼4% respectively.

**Figure 3:**
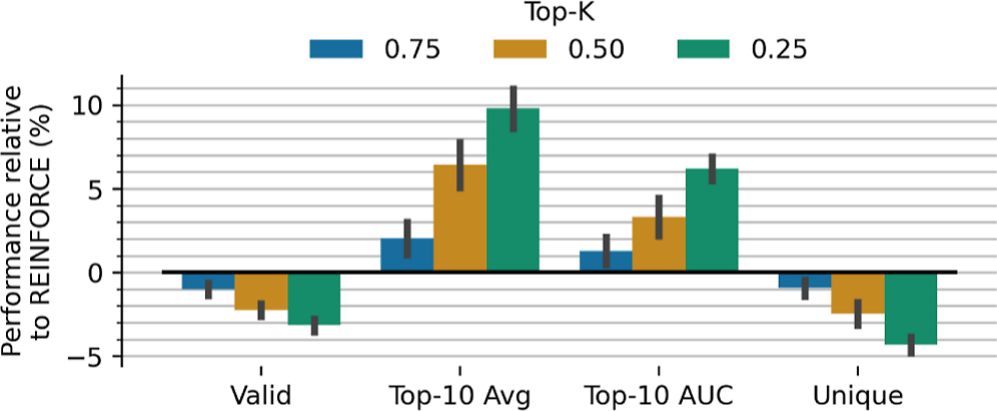
Effect of different top-*k* on-policy subset values (hill-climbing) compared to REINFORCE (which corresponds to top-*k* = 1). Decreasing subset sizes considerably increases effectiveness and efficiency at smaller relative cost to validity and uniqueness.

#### Experience Replay

We explored several experience replay configurations to augment the learning process with off-policy data. Molecules generated by the agent with the highest rewards are stored in a replay buffer and two buffer sampling methods are evaluated to augment on-policy data: sampling buffer molecules uniformly or proportionally to their reward. Additionally, we consider two different sizes for the replay buffer (100 and 500 molecules) and two different batch sizes for sampled buffer data (10 and 20). The results, displayed in Figure 4, indicate that using replayed off-policy data consistently enhances effectiveness (up to ∼10%) and sample efficiency (up to ∼6%), while having no effect on the number of valid molecules generated and limited effect on exploration as measured by the proportion of unique molecules. Prioritized sampling leads to larger performance increases, however, uniform sampling marginally lessens the effect of decreased uniqueness. We find that, out of the configurations tested, a buffer size of 100, batch size of 20 and proportional sampling maximizes both performance and efficiency.

**Figure 4:**
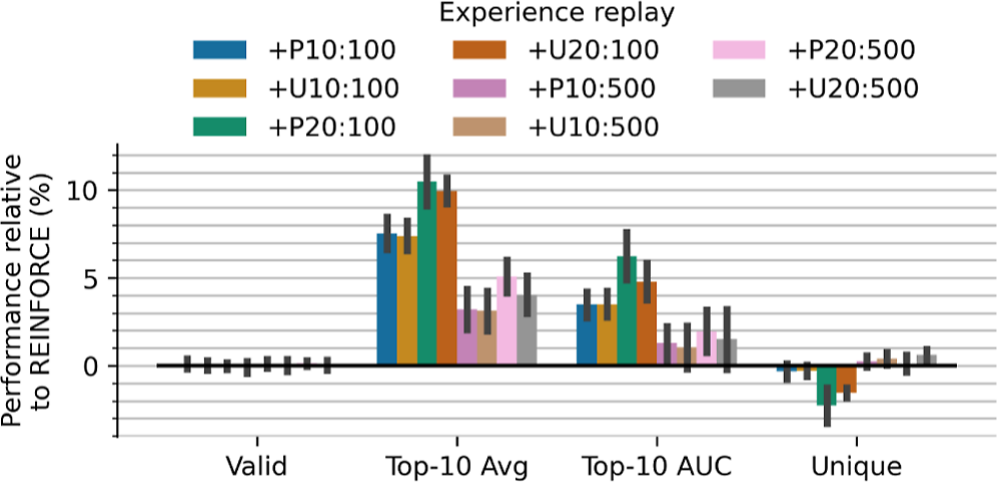
Effect of different experience replay sampling strategies compared to REINFORCE with no experience replay. The initial letter indicates the sampling type: prioritized proportional to the molecule’s reward (P) or uniform (U). The first number after the letter represents the replay batch size, and the final number indicates the experience replay buffer size. Most experience replay strategies increase effectiveness and efficiency at no cost to validity or uniqueness, with smaller replay buffers having a greater effect.

#### Reward Exponent

The new reward shaping mechanism proposed here introduces a tune-able reward exponent α. Note that REINVENT uses a fixed exponential function with a power of 2. To better understand how this exponentiation affects the optimization process, we experimented with several exponent values. Figure 5 shows that increasing the exponent value consistently improves the effectiveness and efficiency up to ∼12% and ∼9% before saturating at an exponent of 5, beyond which no further benefit is observed. Although this reward shaping has a minimal impact on the proportion of valid molecules, it does correspondingly reduce observed exploration, reducing the proportion of unique molecules generated up to ∼9%.

**Figure 5:**
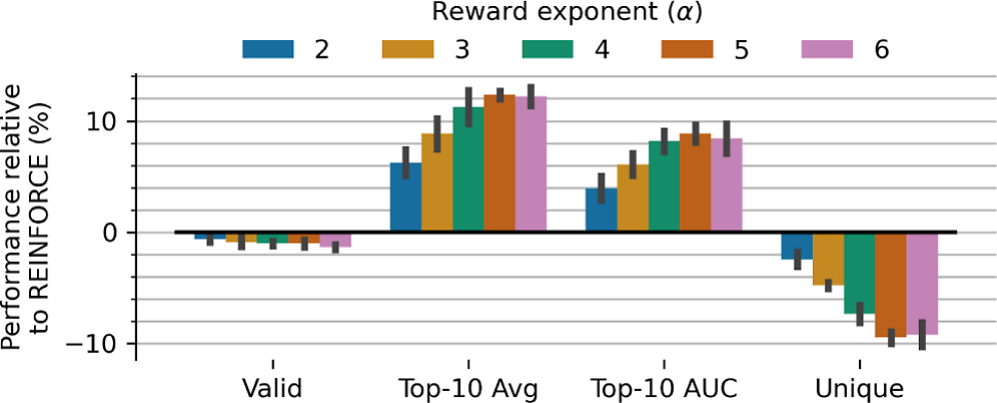
Effect of different reward exponent values compared to REINFORCE (which corresponds to a value of 1). Increasing exponent values that steepen the return gradient lead to increased effectiveness and efficiency at a cost to exploration.

#### Learning Rate

The learning rate of the optimizer could also afford more efficient learning analogous to the update step size in the REINFORCE policy. Figure 6 shows the effect of increasing the learning rates from the default 1 × 10^–4^, as well as annealing the learning rate over the first half of optimization back to the default 1 × 10^–4^. We see that increasing the learning rate to 5 × 10^–4^ increases efficiency but only up to ∼5%, but further increases lead to a decrease in efficiency. Expectedly, all increases result in significantly less exploration as much as ∼26%. Annealing the learning rate from 5 × 10^–4^ to 1 × 10^–4^ does ameliorate the collapse in exploration to ∼10% for an efficiency gain of ∼4%. Overall, this limited set of changes in learning rate does not seem to provide noteworthy performance gains.

**Figure 6:**
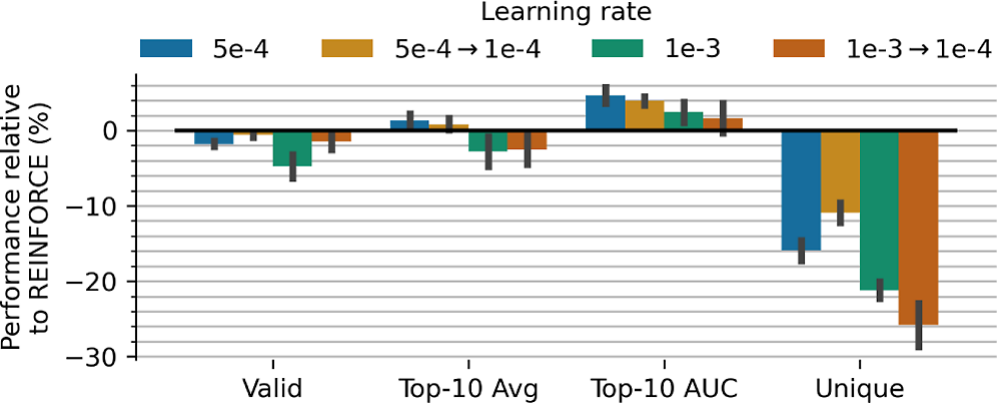
Effect of different learning rates compared to REINFORCE (where the default learning rate is 1 × 10^–4^). The arrow represents the cosine annealing from 5 × 10^–4^ to 1 × 10^–1^ and from 1 × 10^–3^ to 1 × 10^–4^. Higher learning rates show marginal benefit in learning efficiency at a high cost to exploration.

#### Prior Regularization

Regularizing the agent policy to the prior policy can not only avoid catastrophic forgetting but also ensure important aspects of the prior policy are maintained such as the property and chemistry distribution of the training set. In addition to regularization via our proposed reward shaping mechanism, we tested the KL divergence between the agent and prior log-likelihoods. We explore various values for the regularization coefficient σ in our proposed reward reshaping mechanism, as well as different KL coefficients (λ_KL_), illustrated in Figure 7. Note that we did not test different σ for the REINVENT as this does not independently control regularization, but also the scale and gradient of the return landscape. As these parameters more directly relate to the quality of chemistry output, we additionally show the more “chemistry-aware” metrics that also measure the quality of chemistry. Most notably, we observed that the addition of our reward shaping positively impacts the validity of proposed molecules up to ∼12% but does decrease the diversity of the output by up to ∼20%: likely due to the constraint of prior likelihood put on the agent, limiting freedom of exploration. Meanwhile, the KL divergence loss term also increases chemical validity up ∼18% and increases exploration up to ∼12%. This effect is likely due to the KL term penalizing diverging *distributions* of actions at each time step relative to the prior which will have higher entropy than a focused agent. Whereas, any strategy that only accounts for the overall likelihood does not model such distributional changes. However, both strategies lead to a small drop in effectiveness and efficiency. These results show that in this case the KL divergence is more beneficial to regularize prior policy than reward shaping with prior log-likelihood values.

**Figure 7:**
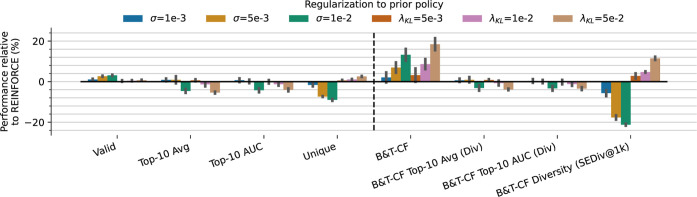
Effect of different policy regularization strategies and parameters, including different σ for the proposed reward shaping and different λ_KL_ coefficients for regularization by KL divergence. Increasing coefficients of both strategies improve validity, more profoundly observed by the “chemistry-aware” metrics on the right. However, the proposed reward shaping correspondingly decreases exploration, whereas KL divergence correspondingly increases exploration.

#### Exploration Techniques

Most of the techniques analyzed so far improve optimization ability but often at the cost of uniqueness, our proxy for exploration. Here, we test strategies to increase agent exploration on the more rigorous “chemistry-aware” metrics including exploration measured by chemical diversity. We considered four strategy options: Maximizing the entropy (ENT), penalizing highly likely agent sequences (ALL), two diversity filters (DF) that either penalize the repetition of molecules, or that penalize molecules similar to those generated previously, and finally a random network distillation (RND) bonus exploration reward. We test ENT, ALL, and RND with two different coefficients (λ) in the loss function (see eqs 6–8). Figure 8 shows that all strategies except the diversity filters increase exploration via B&T-CF Diversity up to ∼18% with RND. However, all strategies except for diversity filters do this at the cost of validity (up to ∼28%), effectiveness (up to ∼12%), and efficiency (up to ∼12%). Higher values of λ_ENT_ and λ_ALL_ were tested but led to considerably worse performance (see Section SD). We find that RND provides the best trade-off in diversity increased vs validity dropped, which may be mitigated by combination with policy regularization strategies. Furthermore, the diversity filters have little impact on performance in this benchmark, due to the short budget of 10,000 molecules not filling memory buffers enough to penalize rewards and affect exploration. We refer the reader to other studies of diversity filters^14,27,29^ for a more thorough investigation of this strategy. We also propose that REINFORCE is already reasonably explorative and these strategies are more beneficial when combined with the other extensions to counter the decreases in exploration observed.

**Figure 8:**
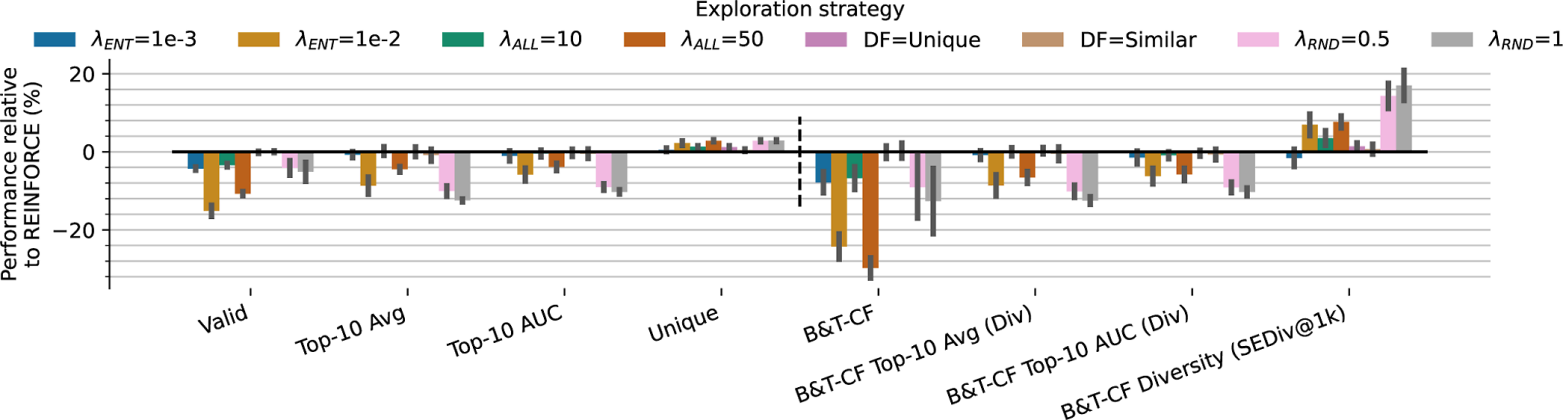
Effect of different exploration strategies and parameters, including an entropy loss term (ENT), a high agent likelihood penalty (ALL), two diversity filters (DF), and random network distillation (RND). Note that all strategies increase exploration measured by B&T-CF diversity to varying degrees, but typically come at the cost of decreased validity, effectiveness and efficiency.

### Optimizing REINFORCE

The extensions to REINFORCE individually tested here are likely not orthogonal and may be anticorrelated. To search for an optimal combination of hyperparameters, we conducted a hyperparameter search based on the most promising extensions observed (see Section SC). We tested a random subset of 1000 configurations out of a combinatorially possible 7,464,960 and approximated performance with two tasks from the MolOpt benchmark. The two tasks selected were “Osimertinib MPO” and “Median molecules 2” that most correlated with overall benchmark performance. Where the best-performing configuration was the one with the highest Top-10 AUC as proposed in the original publication.

The configuration with the best performance observed during hyperparameter optimization we call ACEGEN_MolOpt_, with the corresponding results shown in Table 1. This configuration shows state-of-the-art performance in effectiveness (Top-10 Avg) and efficiency (Top-10 AUC). Although a Bonferroni corrected Wilcoxon signed-rank test on the aggregated benchmark results only showed significant improvement in validity (*p* = 0.002 compared to REINVENT-MolOpt), an analysis at the task level with a TukeyHSD considering all replicates revealed that ACEGEN_MolOpt_ significantly outperformed REINVENT-MolOpt at several tasks in Top-10 Avg and Top-10 AUC (Figure S5). Similar to REINVENT_MolOpt_, ACEGEN_MolOpt_ particularly suffers from low exploration and low B&T-CF validity. In the event that the scoring function(s) perfectly describes chemical desirability, this is not a problem. Moreover, this configuration was identified by searching only ∼0.01% of the combinatorial space. Therefore, it is likely a better-performing configuration exists. We make the hyperparameter search scripts available if further search of this space is of interest to researchers.

**Table 1:** Performance Comparison between Proposed ACEGEN RL Algorithms and Baseline Algorithms on the MolOpt Benchmark^a^

	references				ours	
metric	REINFORCE	REINVENT	REINVENT-MolOpt	AHC	ACEGEN_practical_	ACEGEN_MolOpt_
valid	21.77 ± 0.03	21.79 ± 0.02	21.74 ± 0.05	21.45 ± 0.02	22.03 ± 0.03	**22.17** ± **0.04**
Top-10 Avg	15.85 ± 0.10	15.67 ± 0.11	17.43 ± 0.17	16.09 ± 0.10	16.82 ± 0.21	**17.63** ± **0.13**
Top-10 AUC	13.67 ± 0.07	13.55 ± 0.08	15.65 ± 0.14	13.91 ± 0.08	14.27 ± 0.12	**15.94** ± **0.09**
unique	22.28 ± 0.10	22.67 ± 0.04	13.68 ± 0.31	**22.68** ± **0.07**	18.21 ± 0.30	10.39 ± 0.34
B&T-CF	14.34 ± 0.18	**14.70** ± **0.12**	7.00 ± 0.27	13.77 ± 0.12	13.30 ± 0.25	6.18 ± 0.27
B&T-CF Top-10 Avg (Div)	14.95 ± 0.12	14.79 ± 0.13	16.06 ± 0.16	15.25 ± 0.10	15.50 ± 0.20	**16.07** ± **0.18**
B&T-CF Top-10 AUC (Div)	12.91 ± 0.06	12.81 ± 0.08	14.61 ± 0.13	13.11 ± 0.08	13.28 ± 0.14	**14.72** ± **0.14**
B&T-CF diversity (SEDiv@1k)	17.39 ± 0.15	**18.23** ± **0.11**	10.10 ± 0.27	17.55 ± 0.10	15.69 ± 0.16	12.54 ± 0.25

a ACEGEN_practical_ provides a hand-picked configuration aimed to balance validity, effectiveness, efficiency and exploration; this outperforms its corresponding baseline AHC. ACEGEN_MolOpt_ provides a hyperparameter optimized configuration for the MolOpt benchmark focusing on effectiveness and efficiency, outperforming its corresponding baseline REINVENT_MolOpt_.

On the other hand, more often than not scoring functions do not perfectly describe and account for chemical desirability. In this case, we hand-selected a configuration we call ACEGEN_practical_ to better maintain chemical validity and exploration, for which the hyperparameters are shown in Section SB. This configuration achieves a balance across all metrics. Maintaining similar B&T-CF validity to AHC but improving markedly in effectiveness and efficiency.

### Case Study: Optimizing Putative JNK3 Binding Affinity via Boltz2

To better assess the capability of the ACEGEN_MolOpt_ RL configuration (now on referred to as ACEGEN) we tested its ability to generate molecules guided by frontier binding affinity models.^20^ This was also done within a practical evaluation budget of 10,000 molecules and compared to SynFlowNet^35^ as the baseline model used by Passaro et al.^20^ SynFlowNet is a GFlowNet generative model that is constrained to synthesizable chemical space, hence increasing practicality for industrial drug discovery but also restricting chemical space explored. Figure 9 shows that ACEGEN is able to optimize Boltz2 binding affinity well within the budget, which SynFlowNet does not manage to achieve. This is not especially surprising considering Passaro et al. evaluated up to 400,000 molecules until policy convergence. As SynFlowNet is restricted to synthesizable chemical space, we expected more molecules to be solved by AiZynthFinder.^36^ However, we found the opposite, that proportionally ACEGEN had more molecules with successfully identified synthetic routes throughout optimization. We hypothesize that this is likely due to chemical space biases and alignment between the ChEMBL pretrained CLM and AiZynthFinder, compared to SynFlowNet which uses Enamine building blocks and reaction sets. However, it is encouraging that ACEGEN-proposed designs are also predicted to be synthesizable. For examples of molecules with predicted solved or unsolved synthetic routes see Figures S6 and S7. We did not observe that molecules from either model, or those with better estimated binding affinity were more close to known JNK3 ligands. This suggests that the models are probing novel chemical spaces relative to JNK3 ligands. Overall, ACEGEN is much more capable of learning than SynFlowNet for this particular task and budget, and remains in desirable chemical spaces as measured by QED^37^ and SAScore.

**Figure 9:**
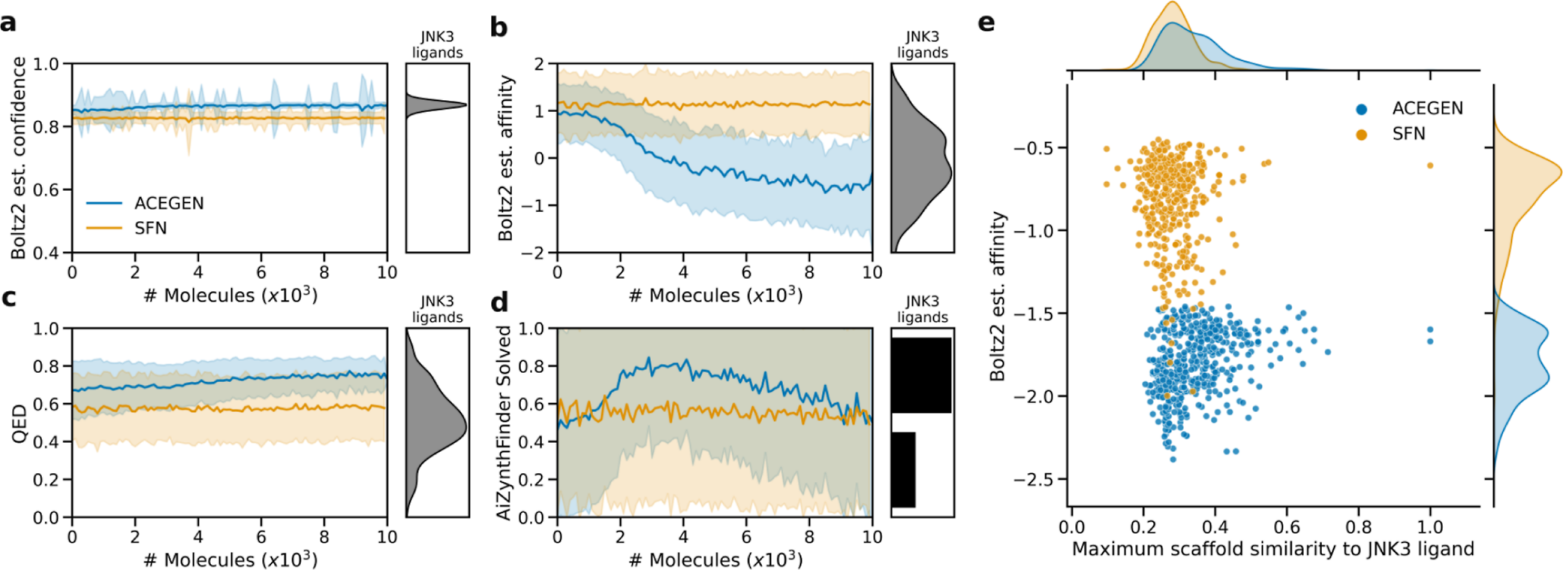
Comparison between ACEGEN and SynFlowNet generated molecules when optimizing the Boltz2 binding affinity of JNK3. These experiments were repeated 5 times with the mean and standard deviation shown. (a) The binding confidence remains high during learning. (b) The affinity improves (decreases) as ACEGEN learns, however, SynFlowNet does not improve within the budget. (c) The QED is improved more so by ACEGEN. (d) Proportion synthesizable as determined by finding a synthetic route with AiZynthFinder. Reference values for known JNK3 ligands are shown for each property Either solved = 1, or not solved = 0. (e) Distribution of estimated binding affinity and scaffold similarity (Tanimoto similarity of Bemis–Murcko scaffolds by ECFP4 fingerprints) to JNK3 ligands of the top 500 molecules from each model. Neither model generated very similar molecules to known JNK3 ligands.

A pivotal challenge in finding kinase inhibitor drug candidates is selectivity, due to highly conserved ATP-binding sites across protein kinases. Allosteric ligands targeting protein pockets show particular promise in identifying more selective drugs.^38^ Therefore, we ran an additional experiment to optimize the Boltz2 binding affinity while additionally cofolding ATP into the orthosteric pocket, encouraging the model to fold putative ligands elsewhere in potential allosteric pockets. Figure 10 shows that learning to optimize the estimated binding affinity is slower but possible, while still maintaining high confidence, and desirable QED and SAScore property value ranges. Likewise, the top 500 molecules (top 100 from each experimental repeat) do not bare close resemblance to known JNK3 ligands—which are mostly orthosteric, ATP-competitive ligands. To estimate kinase selectivity of the resulting molecules, we estimated the binding probability of each molecule to 430 different kinases across the kinome. This was done by utilizing the publicly available random forest models trained to classify ligands taken from ChEMBL and PubChem for each kinase at a concentration of 10 μM.^39^ Figure 11 confirms that the de novo molecules targeting allosteric sites have much improved estimated selectivity profiles than known JNK3 ligands. On the other hand, the ATP-competitive de novo molecules have much worse estimated selectivity profiles than known JNK3 ligands. Therefore, ATP-competitive de novo molecules appear to contain pan-kinome chemotypes which may be influenced by Boltz2 encouraging general kinase ligand features as opposed to, JNK3 specific features.

**Figure 10:**
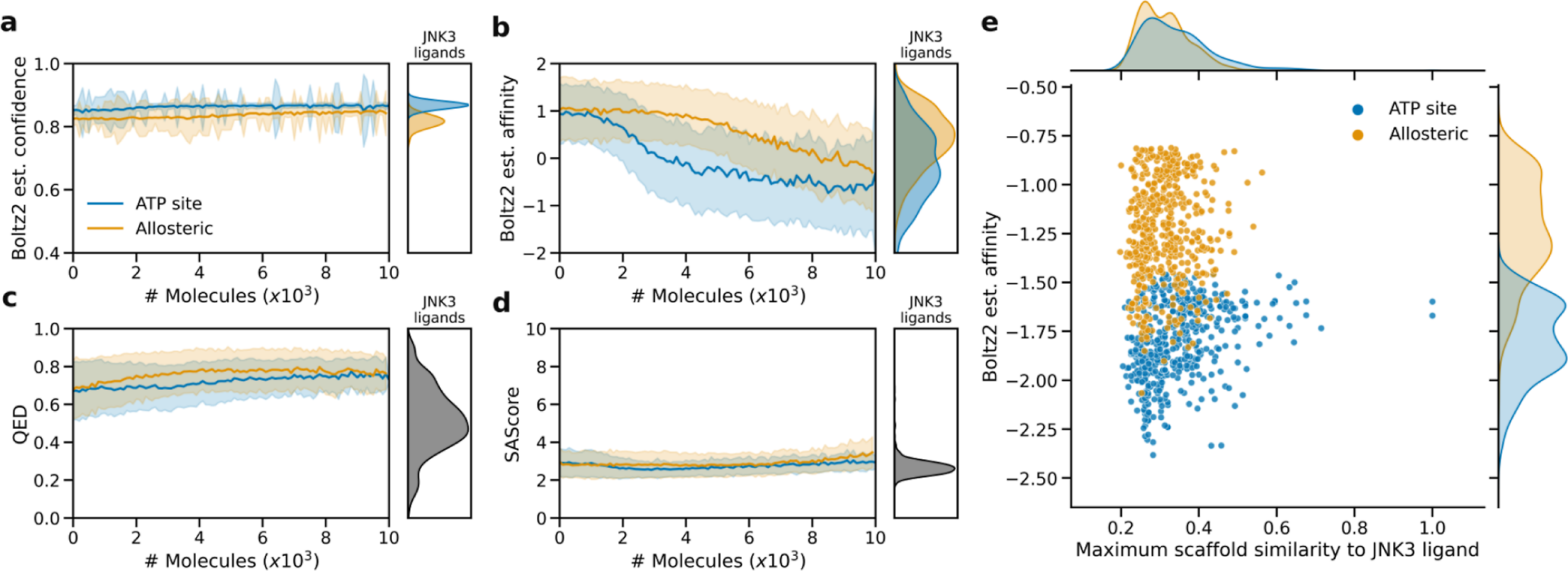
Comparison between optimizing Boltz2 binding affinity in the ATP site or an allosteric site by preoccupation of ATP in the ATP site. These experiments were repeated 5 times with the mean and standard deviation shown. (a) The binding confidence remains high during learning. (b). Estimated binding affinity improves at a slower rate for allosteric binding. (c) The QED remains high for both tasks. (d) Synthesizability score (SAScore) remains equally low for both tasks. Reference values for known JNK3 ligands are shown for each property. (e) Distribution of estimated binding affinity and scaffold similarity (Tanimoto similarity of Bemis–Murcko scaffolds by ECFP4 fingerprints) to JNK3 ligands of the top 500 molecules from each task. Neither task generates very similar molecules to known JNK3 ligands.

**Figure 11:**
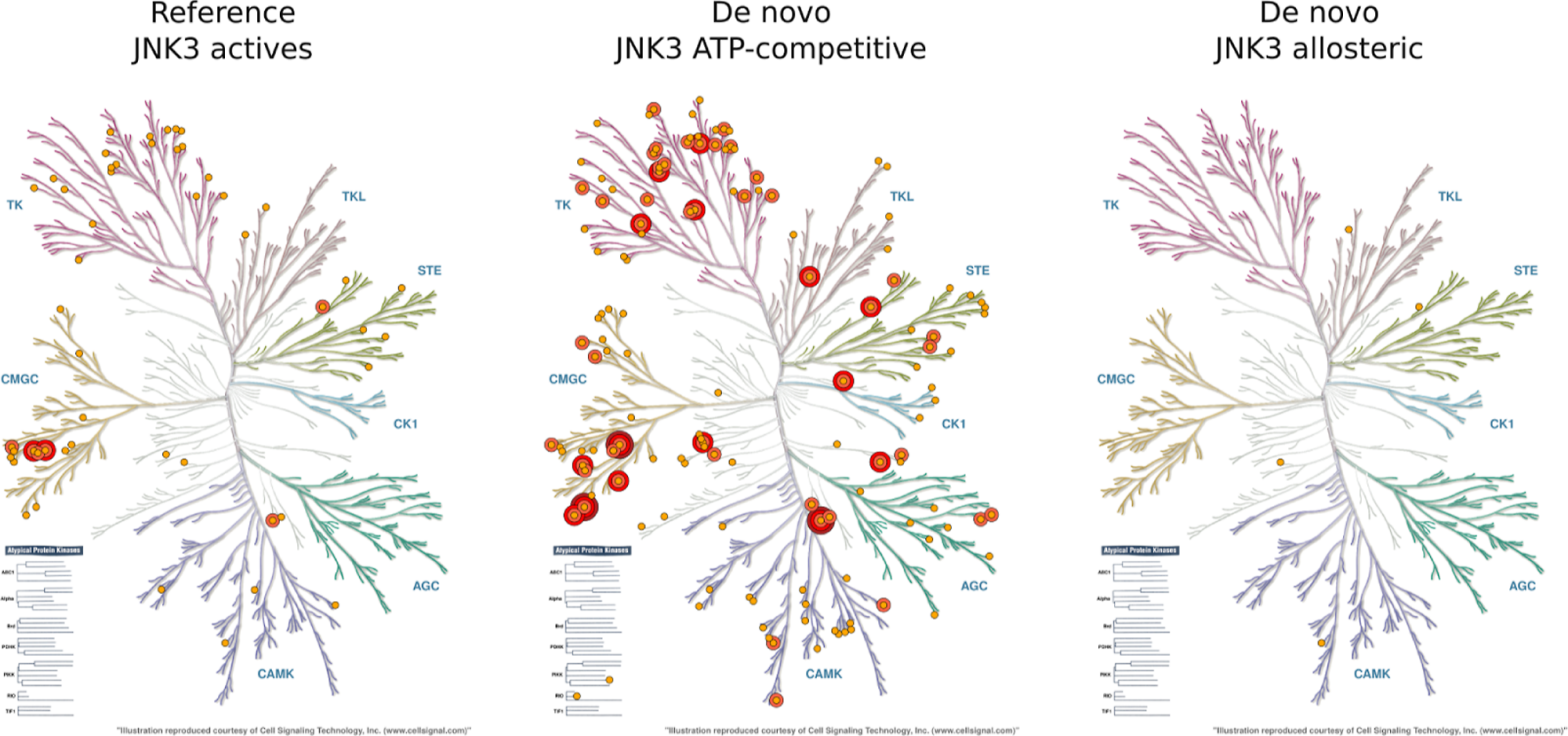
Estimated kinase selectivity profile for JNK3 ligands, ATP-competitive de novo molecules, and Allosteric de novo molecules. Kinases are annotated according to the percent of ligands estimated active at a concentration of 10 μM (dark red >80%, red >60%, salmon >40%, orange >20%). Figures were generated using KinMap.^40^

Inspecting the cofolded structures showed that the vast majority of putative allosteric ligands were cofolded into the docking site for ERK or FXF (DEF).^41^ This is a substrate-binding site located between the MAPK insert and αG helix that recognizes conserved FXF motifs required for binding by some downstream substrates, although better characterized in ERK^41^ this motif has been implicated in MAPK phosphatases determining JNK1 recognition.^42^ However, there is no publicly available structure of a small molecule bound to the DEF site of JNK3, there only exists one of the closely related JNK1 kinase.^43^ The best de novo compounds for each pocket are shown in Figure 12. The best allosteric compound is projected to bind with a dichloro napthalene moiety in the shallow lipophilic pocket forming hydrophobic interactions with Tyr295, Thr291, Val292, and Ile267 with an additional halogen bond to the backbone carbonyl of Ile233. Conversely, the rest of the compound is polar with a piperazine core and 2,4-dicyano-1,3-diaminobenzene forming a hydrogen bond with Thr214. Although the 2,4-dicyano-1,3-diaminobenzene structure is likely not synthetically accessible or stable in this case, many other generated compounds share this amphiphilic nature with the piperazine core commonly appearing (see Section SE.3).

**Figure 12:**
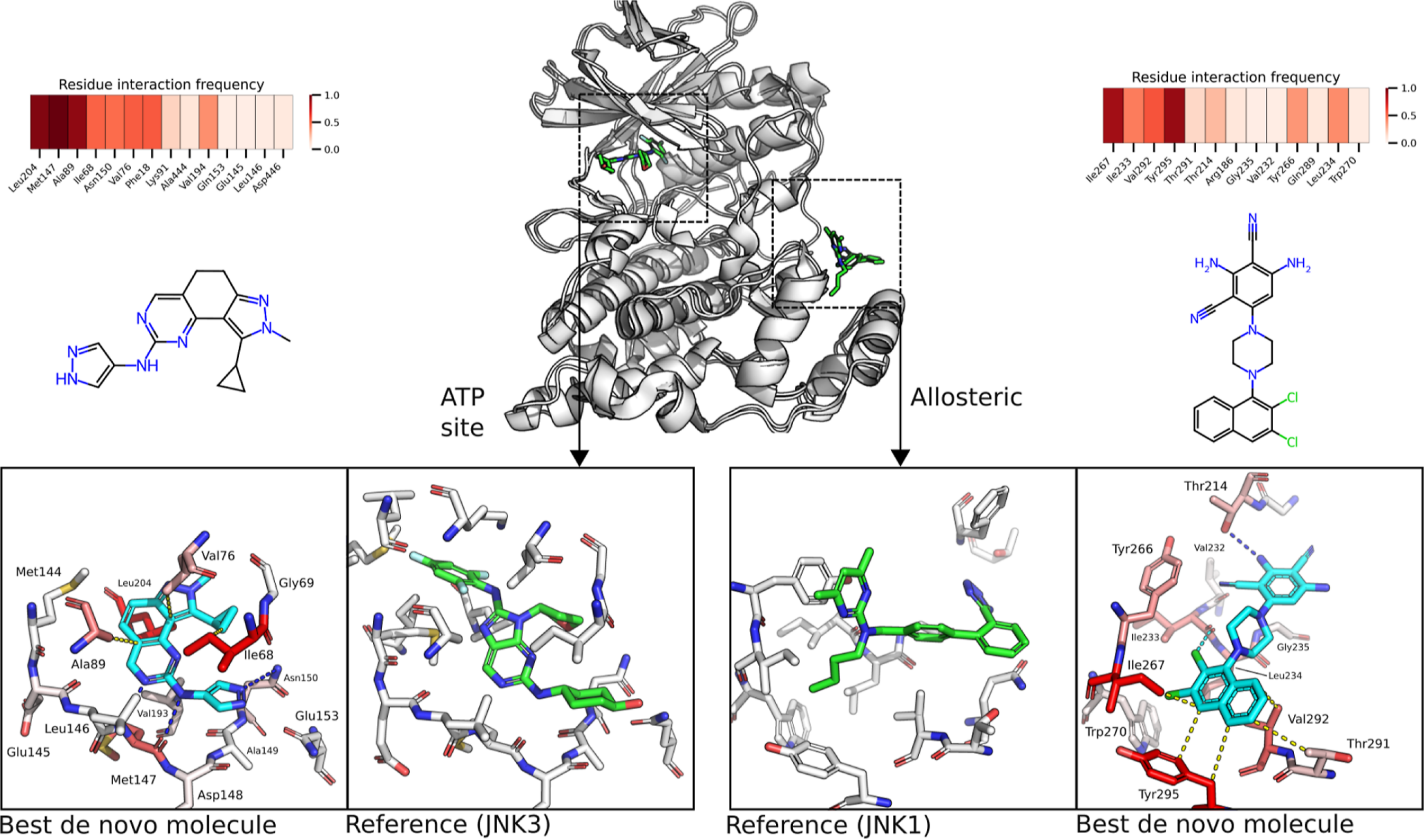
De novo molecule with the best Boltz2 binding affinity for each ATP-competitive and allosteric experiment, alongside reference structures. JNK3 reference is the CC-930 drug candidate (PDB: 3TTI).^44^ There are no reported JNK3 small-molecule allosteric structures, hence, the reference structure is JNK1 isoform bound to an allosteric inhibitor (PDB:3O2M)^43^ in the DEF site.^41^

To further assess allosteric binding from Boltz2, we selected a representative subset of 10 compounds across four Boltz2-estimated binding affinity ranges and evaluated their accuracy using an orthogonal physics-based method: absolute binding free energy (ABFE).^45^ Unlike relative binding free energy methods, which are optimal for congeneric series,^20^ ABFE provides a rigorous and transferable measure of binding strength by estimating the free energy difference between bound and unbound states of a ligand. In this work we use absolute free energy perturbation (AFEP) formalism as implemented in Schrödinger FEP + package. As shown in Figure 13, Boltz2 estimated affinities correlate with AFEP derived binding affinities. Binned affinities show a significant variance across means as tested by a one-way ANOVA (*p* = 0.0012), and nonbinned affinities report a Pearson correlation of 0.7. In the absence of experimental data, these results indicate strong alignment between Boltz2 and AFEP, providing additional confidence in the use of this approach to search for new protein pockets and optimizing chemical matter binding to them.

**Figure 13:**
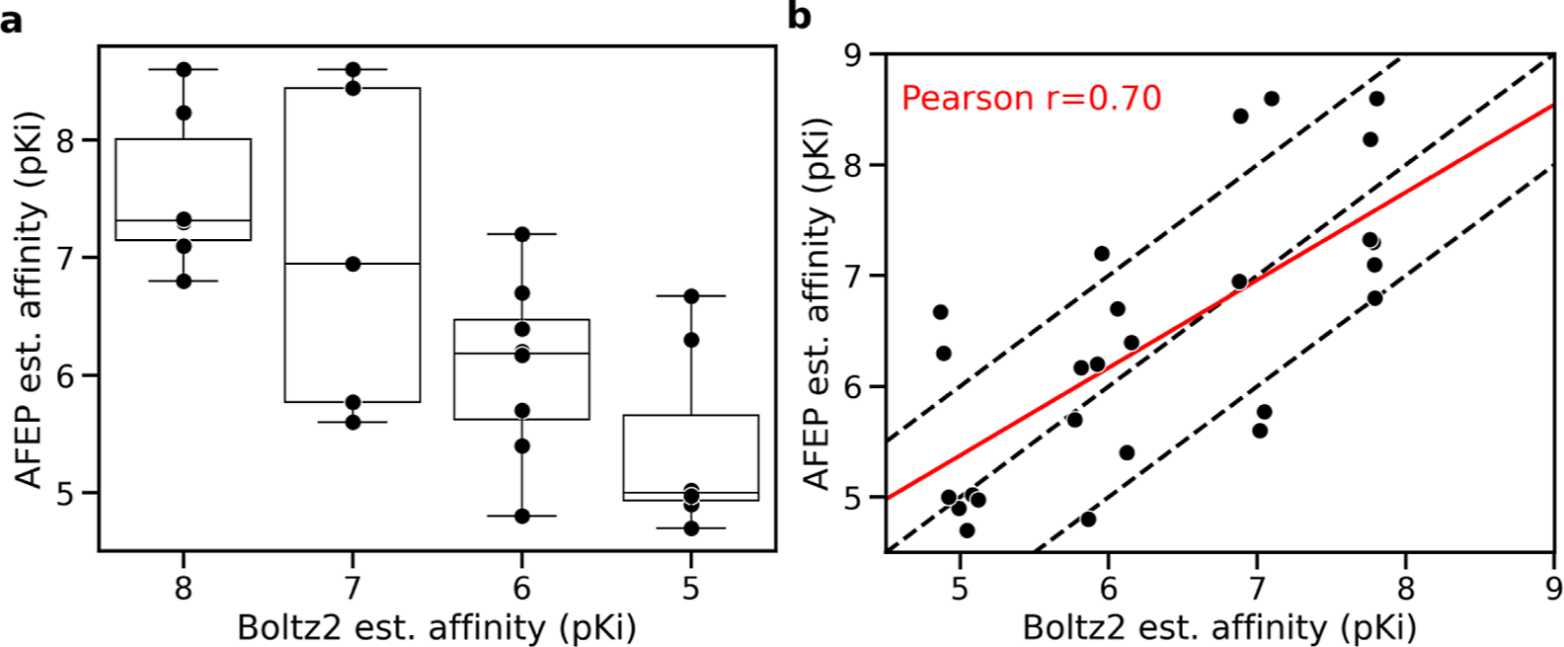
Correlation between Boltz2 estimated pKi and ABFE estimated pKi. (a) Binned affinities show a positive stepwise correlation, where a one-way ANOVA test reports significant variance between the means (*p* = 0.0012). (b) The nonbinned correlation between Boltz2 estimated affinity and AFEP with a reported Pearson correlation of 0.7.

## Conclusion

In this work, we revisited the REINFORCE algorithm, investigating additional extensions stemming from RL theory for de novo molecule generation with chemical language models. We investigated extensions believed to improve the validity, effectiveness and efficiency of learning, and exploration. Moreover, we uncovered a new reward-shaping mechanism to provide more control over the trade-off between optimization efficiency and prior policy regularization. Rebuilding REINFORCE by combining the investigated extensions demonstrates capacity for improvement of this algorithm demonstrated on the molecular optimization benchmark: showing state-of-the-art performance according to the metric of AUC Top-10, proposed by Gao et al., as well as excellent performance on our suite of metrics that additionally include chemistry. This performance is measured on a benchmark that focuses on exploitation, however, when tested on a case study to find putative allosteric JNK3 ligands, we also observed strong performance. Our version of the REINFORCE algorithm vastly outperforms the baseline model SynFlowNet in our sample efficiency scenario, and is able to optimize Boltz2 binding affinity considerably while maintaining drug-like property ranges of de novo molecules. Principally, we hope the results here act as a guide for implementing RL with CLMs. We make all the RL extensions, scripts for hyperparameter optimization, and the other CLMs mentioned above available in ACEGEN.

## Supplementary Material



## Data Availability

All software used in this manuscript is freely available open-source under an MIT license. The RL configurations tested and used are incorporated into ACEGEN, available on GitHub (https://github.com/Acellera/acegen-open). The parameters of the pretrained model used in this work are also available in ACEGEN repository. Benchmarking was conducted using MolScore which is available on GitHub (https://github.com/MorganCThomas/MolScore) and in the Python Package Index (https://pypi.org/project/MolScore/).

## Notes

Methods, figures, and tables referenced in the text (PDF)
