# Supplementary material for: REINFORCE-ING Chemical Language Models for Drug Discovery
Source: J Chem Inf Model. 2025 Nov 16;65(23):12752–63. doi: 10.1021/acs.jcim.5c02053 (PMC12690592; doi:10.1021/acs.jcim.5c02053)
Supplement: Supplementary file 1 [file ci5c02053_si_001.pdf]

Supporting Information: REINFORCE-ING Chemical Language Models for Drug Discovery

Morgan Thomas,[*,†,‡] Albert Bou,[†] Jose Carlos Gómez-Tamayo,[¶] Gary Tresadern,[¶] Mazen Ahmad,[¶] and Gianni De Fabritiis[*,§,†,‖]

†*Computational Science Laboratory, Universitat Pompeu Fabra, Barcelona Biomedical Research Park (PRBB), C Dr. Aiguader 88, 08003 Barcelona, Spain*

‡*Khalifa University of Science and Technology, Abu Dhabi, UAE*

¶*In Silico Discovery, Janssen Research & Development, Janssen Pharmaceutica N. V., Turnhoutseweg 30, B-2340 Beerse, Belgium*

§*Institució Catalana de Recerca i Estudis Avançats (ICREA), Passeig Lluis Companys 23, 08010 Barcelona, Spain*

‖*Acellera Labs, C Dr Trueta 183, 08005, Barcelona, Spain*

E-mail: morganthomas263@gmail.com; g.defabritiis@gmail.com

Contents

A Reward shaping landscape **S-3**

B Baseline algorithm hyperparameters **S-5**

C ACEGEN hyperparameter optimization **S-8**

D Performance of individual REINFORCE extensions **S-9**

E Optimizing Boltz2 estimated binding affinity **S-12**

E.1 Boltz2 reward function S-12

E.2 SynFlowNet S-16

E.3 De novo generated compounds S-20

E.4 Absolute binding free energy S-24

References **S-29**

A Reward shaping landscape

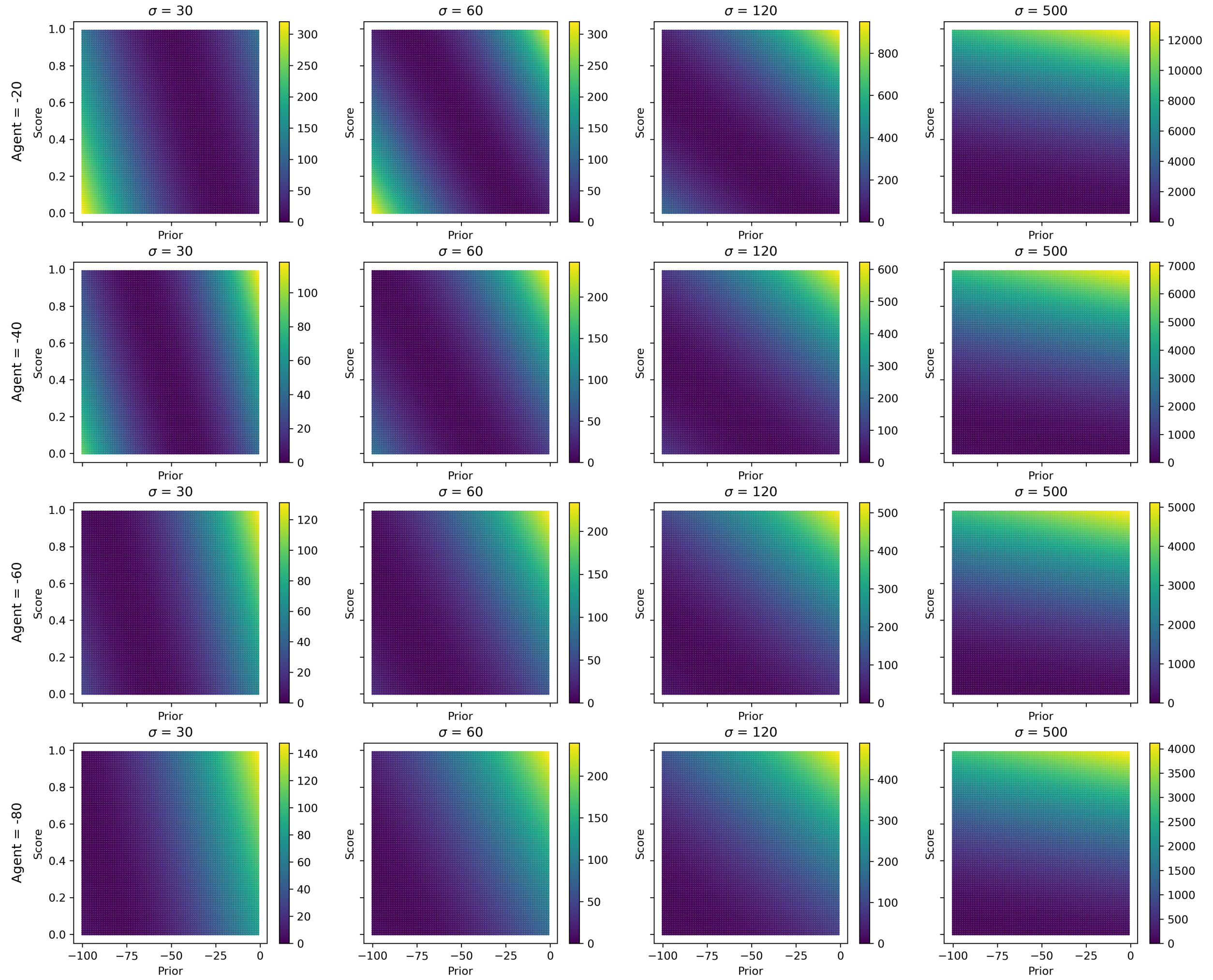

Figure S1: REINVENT reward shaping landscape depending on input reward (Score), Agent log-likelihood (Agent), Prior log-likelihood (Prior) and sigma (σ). Color reflects the reshaped reward.

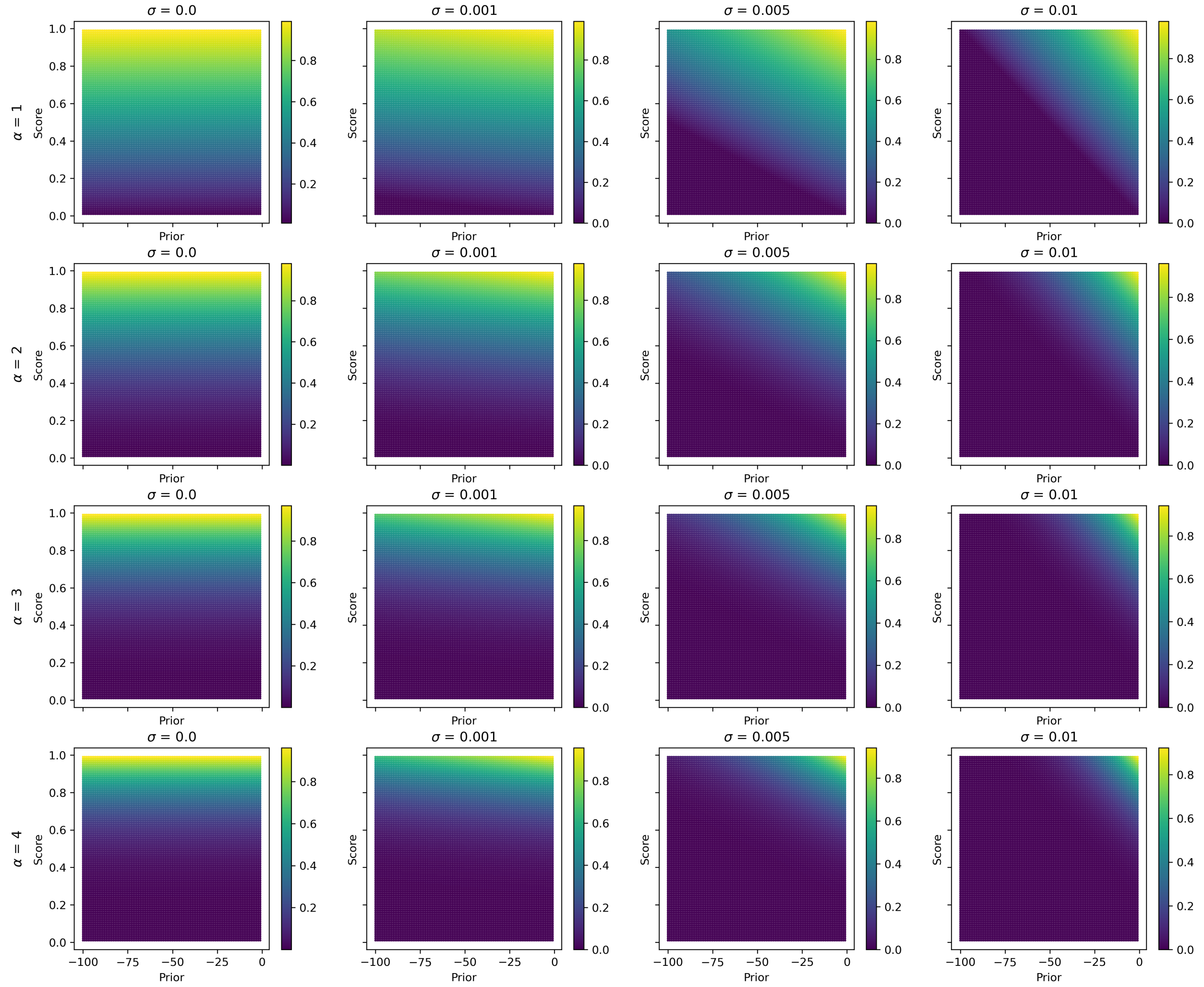

Figure S2: ACEGEN reward shaping landscape depending on input reward (Score), alpha (α), Prior log-likelihood (Prior) and sigma (σ). Color reflects the reshaped reward.

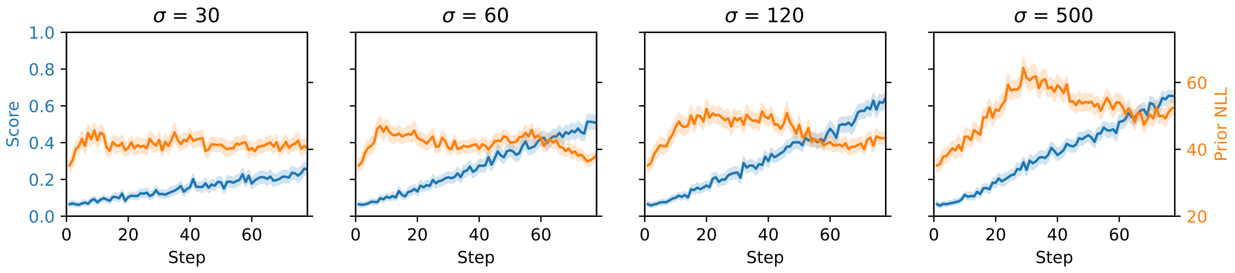

Figure S3: Performance of REINVENT at different values of σ on the JNK3 MolOpt benchmark task. On the left y-axis (blue) is the JNK3 score during training step, while on the right y-axis (orange) is the negative log-likelihood (NLL) (lower is more likely, and hence better) of molecules according to the prior policy. Variables are measured during RL training steps until 10,000 molecules have been evaluated.

B Baseline algorithm hyperparameters

Table S1: Hyperparameters for REINFORCE.

Hyperparameter	Value
num_envs	128
total_smiles	10,000
model	GRU (embedding of size 256 + 3 layer GRU of size 512 + MLP)
lr	0.0001
experience_replay	True
replay_buffer_size	100
replay_batch_size	10

Table S2: Hyperparameters for REINVENT.

Hyperparameter	Value
num_envs	128
total_smiles	10,000
model	GRU (embedding of size 256 + 3 layer GRU of size 512 + MLP)
lr	0.0001
experience_replay	True
replay_buffer_size	100
replay_batch_size	10
sigma	120

Table S3: Hyperparameters for REINVENT$_{MolOpt}$.

Hyperparameter	Value
num_envs	64
total_smiles	10,000
model	GRU (embedding of size 256 + 3 layer GRU of size 512 + MLP)
lr	0.0005
experience_replay	True
replay_buffer_size	100
replay_batch_size	24
sigma	500

Table S4: Hyperparameters for AHC.

Hyperparameter	Value
num_envs	128
total_smiles	10,000
model	GRU (embedding of size 256 + 3 layer GRU of size 512 + MLP)
lr	0.0001
experience_replay	True
replay_buffer_size	100
replay_batch_size	10
sigma	60
topk	0.5

Table S5: Hyperparameters for $\text{ACEGEN}_{Practical}$.

Hyperparameter	Value
num_envs	128
total_smiles	10,000
model	GRU (embedding of size 256 + 3 layer GRU of size 512 + MLP)
lr	0.0001
experience_replay	True
replay_batch_size	10
replay_buffer_size	100
replay_sampler	uniform
sigma	0.005
topk	0.5
alpha	5
baseline	mab

Table S6: Hyperparameters for ACEGEN$_{MolOpt}$.

Hyperparameter	Value
num_envs	32
total_smiles	10,000
model	GRU (embedding of size 256 + 3 layer GRU of size 512 + MLP)
lr	0.0001
experience_replay	True
replay_batch_size	50
replay_buffer_size	100
replay_sampler	prioritized
sigma	0.001
topk	0.5
alpha	3
baseline	False

C ACEGEN hyperparameter optimization

Table S7: Hyperparameter search space defined for ACEGEN.

Hyperparameter	Value
num_envs	32, 64, 128, 256
total_smiles	10,000
model	GRU (embedding of size 256 + 3 layer GRU of size 512 + MLP)
lr	0.0001, 0.0005
lr_annealing	False, True
experience_replay	False, True
replay_batch_size	10, 20, 50
replay_buffer_size	100, 500
replay_sampler	uniform, prioritized
sigma	0, 1e-3, 5e-3, 1e-2
kl_coef	0, 5e-3, 1e-2, 5e-2
alpha	1, 2, 3, 4, 5
topk	0.25, 0.5, 0.75, 1.0
baseline	False, mab, loo
entropy_coef	0, 1e-3, 1e-2
likely_penalty	0, 10, 50
rnd_coef	0, 0.5, 1.0

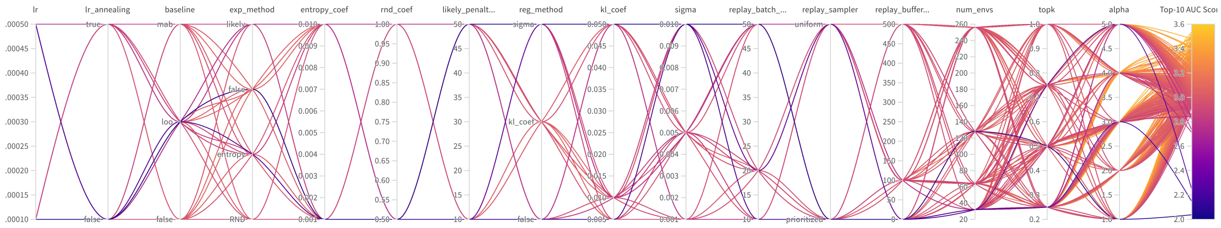

Figure S4: Sweep plot visualizing the effect of hyperparameters on the performance of "Osimertinib MPO" and "Median molecules 2" as measured by Top-10 AUC.

D Performance of individual REINFORCE extensions

Table S8: Effect of different reward baselines including a moving average baseline (MAB) and leave-one-out baseline (LOO) on MolOpt benchmark performance.

	REINFORCE	MAB	LOO
Valid	21.74 ± 0.04	**21.94 ± 0.02**	21.92 ± 0.02
Top-10 Avg	14.74 ± 0.09	15.63 ± 0.11	**15.69 ± 0.10**
Top-10 AUC	13.21 ± 0.07	**13.76 ± 0.07**	13.76 ± 0.09
Unique	**22.35 ± 0.05**	21.66 ± 0.10	21.40 ± 0.11
B&T-CF	**14.15 ± 0.23**	12.90 ± 0.15	12.60 ± 0.17
B&T-CF Top-10 Avg (Div)	13.81 ± 0.09	**14.68 ± 0.10**	14.67 ± 0.13
B&T-CF Top-10 AUC (Div)	12.51 ± 0.07	**12.97 ± 0.06**	12.89 ± 0.07
B&T-CF Diversity (SEDiv@1k)	**17.61 ± 0.18**	15.49 ± 0.15	15.57 ± 0.12

Table S9: Effect of different learning rates including cosine annealing back to REINFORCE default (→) on MolOpt benchmark performance.

	REINFORCE (1e-4)	5e-4	5e-4 → 1e-4	1e-3	1e-3 → 1e-4
Valid	**21.74 ± 0.04**	21.36 ± 0.08	21.61 ± 0.07	20.71 ± 0.25	21.42 ± 0.18
Top-10 Avg	14.74 ± 0.09	**14.93 ± 0.18**	14.86 ± 0.18	14.33 ± 0.18	14.36 ± 0.27
Top-10 AUC	13.21 ± 0.07	**13.83 ± 0.14**	13.73 ± 0.14	13.54 ± 0.14	13.43 ± 0.22
Unique	**22.35 ± 0.05**	18.80 ± 0.27	19.92 ± 0.38	17.62 ± 0.29	16.59 ± 0.44
B&T-CF	**14.15 ± 0.23**	7.36 ± 0.39	9.85 ± 0.52	3.92 ± 0.31	4.83 ± 0.44
B&T-CF Top-10 Avg (Div)	13.81 ± 0.09	**13.98 ± 0.16**	13.92 ± 0.16	13.18 ± 0.18	13.29 ± 0.26
B&T-CF Top-10 AUC (Div)	12.51 ± 0.07	**12.97 ± 0.12**	12.87 ± 0.12	12.52 ± 0.14	12.44 ± 0.23
B&T-CF Diversity (SEDiv@1k)	**17.61 ± 0.18**	11.02 ± 0.38	11.11 ± 0.43	8.30 ± 0.37	8.19 ± 0.42

Table S10: Effect of different top-k ratios on MolOpt benchmark performance.

	REINFORCE	0.75	0.5	0.25
Valid	**21.74 ± 0.04**	21.52 ± 0.03	21.25 ± 0.05	21.06 ± 0.06
Top-10 Avg	14.74 ± 0.09	15.04 ± 0.13	15.69 ± 0.09	**16.18 ± 0.07**
Top-10 AUC	13.21 ± 0.07	13.38 ± 0.07	13.65 ± 0.07	**14.03 ± 0.06**
Unique	**22.35 ± 0.05**	22.15 ± 0.09	21.80 ± 0.11	21.39 ± 0.09
B&T-CF	**14.15 ± 0.23**	12.77 ± 0.26	12.10 ± 0.21	11.66 ± 0.19
B&T-CF Top-10 Avg (Div)	13.81 ± 0.09	14.05 ± 0.12	14.57 ± 0.11	**15.06 ± 0.08**
B&T-CF Top-10 AUC (Div)	12.51 ± 0.07	12.61 ± 0.06	12.79 ± 0.06	**13.13 ± 0.06**
B&T-CF Diversity (SEDiv@1k)	**17.61 ± 0.18**	17.59 ± 0.14	16.95 ± 0.15	15.89 ± 0.14

Table S11: Effect of different experience replay parameters on MolOpt benchmark performance. The initial letter indicates the sampling type: prioritized proportional to the molecule's reward (P) or uniform (U). The first number after the letter represents the replay batch size, and the final number indicates the experience replay buffer size.

	REINFORCE	+P10:100	+U10:100	+P20:100	+U20:100	+P10:500	+U10:500	+P20:500	+U20:500
Valid	21.74 ± 0.04	**21.77 ± 0.03**	21.75 ± 0.03	21.74 ± 0.03	21.72 ± 0.03	21.77 ± 0.03	21.75 ± 0.03	21.77 ± 0.03	21.75 ± 0.03
Top-10 Avg	14.74 ± 0.09	15.85 ± 0.10	15.83 ± 0.06	**16.28 ± 0.09**	16.21 ± 0.10	15.21 ± 0.11	15.20 ± 0.10	15.49 ± 0.14	15.33 ± 0.11
Top-10 AUC	13.21 ± 0.07	13.67 ± 0.07	13.68 ± 0.06	**14.04 ± 0.08**	13.85 ± 0.12	13.38 ± 0.08	13.35 ± 0.07	13.47 ± 0.10	13.41 ± 0.09
Unique	22.35 ± 0.05	22.28 ± 0.10	22.29 ± 0.08	21.85 ± 0.12	22.01 ± 0.08	22.41 ± 0.06	22.45 ± 0.04	22.38 ± 0.07	**22.49 ± 0.03**
B&T-CF	14.15 ± 0.23	14.34 ± 0.18	14.30 ± 0.18	13.83 ± 0.20	14.14 ± 0.18	14.64 ± 0.16	14.55 ± 0.19	14.69 ± 0.15	**14.85 ± 0.18**
B&T-CF Top-10 Avg (Div)	13.81 ± 0.09	14.95 ± 0.12	14.89 ± 0.08	**15.26 ± 0.13**	15.22 ± 0.16	14.26 ± 0.09	14.33 ± 0.13	14.62 ± 0.14	14.42 ± 0.13
B&T-CF Top-10 AUC (Div)	12.51 ± 0.07	12.91 ± 0.06	12.92 ± 0.05	**13.19 ± 0.07**	13.04 ± 0.15	12.67 ± 0.08	12.62 ± 0.07	12.74 ± 0.08	12.65 ± 0.07
B&T-CF Diversity (SEDiv@1k)	17.61 ± 0.18	17.39 ± 0.15	17.43 ± 0.13	17.04 ± 0.13	17.15 ± 0.16	17.72 ± 0.13	**17.82 ± 0.11**	17.62 ± 0.15	17.79 ± 0.13

Table S12: Effect of different α exponent values on MolOpt benchmark performance.

	REINFORCE	2	3	4	5	6
Valid	**21.74 ± 0.04**	21.61 ± 0.03	21.55 ± 0.04	21.53 ± 0.04	21.52 ± 0.04	21.45 ± 0.03
Top-10 Avg	14.74 ± 0.09	15.66 ± 0.17	16.04 ± 0.09	16.39 ± 0.13	**16.56 ± 0.13**	16.54 ± 0.11
Top-10 AUC	13.21 ± 0.07	13.73 ± 0.11	14.02 ± 0.08	14.29 ± 0.08	**14.38 ± 0.08**	14.32 ± 0.08
Unique	**22.35 ± 0.05**	21.80 ± 0.12	21.28 ± 0.13	20.71 ± 0.17	20.24 ± 0.16	20.29 ± 0.16
B&T-CF	**14.15 ± 0.23**	12.75 ± 0.19	12.34 ± 0.17	11.95 ± 0.22	11.62 ± 0.19	11.65 ± 0.21
B&T-CF Top-10 Avg (Div)	13.81 ± 0.09	14.64 ± 0.17	15.00 ± 0.16	15.33 ± 0.11	**15.41 ± 0.16**	15.40 ± 0.12
B&T-CF Top-10 AUC (Div)	12.51 ± 0.07	12.87 ± 0.08	13.12 ± 0.10	13.39 ± 0.09	**13.45 ± 0.09**	13.39 ± 0.08
B&T-CF Diversity (SEDiv@1k)	**17.61 ± 0.18**	17.28 ± 0.14	17.04 ± 0.14	16.93 ± 0.13	16.86 ± 0.17	17.03 ± 0.16

Table S13: Effect of different regularization strategies on MolOpt benchmark performance. Including different σ values for the proposed reward shaping and different λ_{KL} coefficients for regularization by KL divergence.

	REINFORCE	σ=1e-3	σ=5e-3	σ=1e-2	λ_{KL}=5e-3	λ_{KL}=1e-2	λ_{KL}=5e-2
Valid	21.74 ± 0.04	21.97 ± 0.03	22.32 ± 0.02	**22.42 ± 0.01**	21.81 ± 0.02	21.84 ± 0.02	21.89 ± 0.01
Top-10 Avg	14.74 ± 0.09	14.86 ± 0.11	**14.87 ± 0.19**	14.06 ± 0.16	14.84 ± 0.10	14.54 ± 0.10	13.94 ± 0.10
Top-10 AUC	13.21 ± 0.07	**13.30 ± 0.08**	13.23 ± 0.10	12.65 ± 0.09	13.20 ± 0.07	13.04 ± 0.07	12.67 ± 0.10
Unique	22.35 ± 0.05	21.98 ± 0.09	20.71 ± 0.10	20.34 ± 0.11	22.48 ± 0.04	22.60 ± 0.04	**22.93 ± 0.05**
B&T-CF	14.15 ± 0.23	14.44 ± 0.20	15.12 ± 0.12	16.01 ± 0.12	14.59 ± 0.17	15.36 ± 0.12	**16.75 ± 0.06**
B&T-CF Top-10 Avg (Div)	13.81 ± 0.09	13.90 ± 0.09	**13.94 ± 0.15**	13.38 ± 0.16	13.92 ± 0.09	13.71 ± 0.10	13.28 ± 0.11
B&T-CF Top-10 AUC (Div)	12.51 ± 0.07	**12.55 ± 0.06**	12.51 ± 0.07	12.09 ± 0.07	12.48 ± 0.06	12.35 ± 0.06	12.07 ± 0.09
B&T-CF Diversity (SEDiv@1k)	17.61 ± 0.18	16.62 ± 0.14	14.50 ± 0.13	13.87 ± 0.14	18.11 ± 0.13	18.45 ± 0.12	**19.63 ± 0.08**

Table S14: Effect of different exploration strategies on MolOpt benchmark performance. Including different entropy coefficients λ_{ENT}, agent likelihood penalty coefficients λ_{ALL}, diversity filters (DF), and random network distillation coefficients λ_{RND}.

	REINFORCE	λ_{ENT}=1e-3	λ_{ENT}=1e-2	λ_{ENT}=1e-1	λ_{ALL}=10	λ_{ALL}=50	λ_{ALL}=100	DF=Unique	DF=Similar	λ_{RND}=0.5	λ_{RND}=1
Valid	**21.74 ± 0.04**	20.81 ± 0.06	18.46 ± 0.41	4.84 ± 0.12	20.99 ± 0.04	19.41 ± 0.11	18.35 ± 0.10	21.71 ± 0.03	21.74 ± 0.03	20.85 ± 0.11	20.63 ± 0.14
Top-10 Avg	14.74 ± 0.09	14.63 ± 0.14	13.46 ± 0.27	10.72 ± 0.13	**14.77 ± 0.10**	14.08 ± 0.16	13.38 ± 0.12	14.74 ± 0.12	14.61 ± 0.14	13.26 ± 0.14	12.90 ± 0.13
Top-10 AUC	**13.21 ± 0.07**	13.07 ± 0.15	12.44 ± 0.21	10.57 ± 0.13	13.16 ± 0.06	12.70 ± 0.13	12.28 ± 0.09	13.18 ± 0.07	13.15 ± 0.08	12.02 ± 0.10	11.86 ± 0.09
Unique	22.35 ± 0.05	22.46 ± 0.05	22.85 ± 0.12	22.96 ± 0.01	22.64 ± 0.04	22.98 ± 0.00	**23.00 ± 0.00**	22.62 ± 0.03	22.45 ± 0.07	22.98 ± 0.00	22.99 ± 0.00
B&T-CF	14.15 ± 0.23	13.03 ± 0.24	10.71 ± 0.23	2.24 ± 0.04	13.19 ± 0.19	9.93 ± 0.20	7.61 ± 0.19	14.13 ± 0.22	**14.18 ± 0.18**	12.84 ± 0.26	12.34 ± 0.26
B&T-CF Top-10 Avg (Div)	13.81 ± 0.09	13.69 ± 0.17	12.62 ± 0.30	10.13 ± 0.09	13.81 ± 0.10	12.90 ± 0.17	12.27 ± 0.12	**13.85 ± 0.10**	13.74 ± 0.11	12.41 ± 0.12	12.09 ± 0.11
B&T-CF Top-10 AUC (Div)	**12.51 ± 0.07**	12.32 ± 0.19	11.72 ± 0.21	9.99 ± 0.09	12.39 ± 0.06	11.78 ± 0.14	11.41 ± 0.09	12.46 ± 0.06	12.42 ± 0.06	11.36 ± 0.09	11.22 ± 0.08
B&T-CF Diversity (SEDiv@1k)	17.61 ± 0.18	17.34 ± 0.19	19.31 ± 0.10	4.64 ± 0.03	18.41 ± 0.13	18.96 ± 0.11	19.81 ± 0.08	17.86 ± 0.14	17.73 ± 0.14	20.13 ± 0.13	**20.61 ± 0.14**

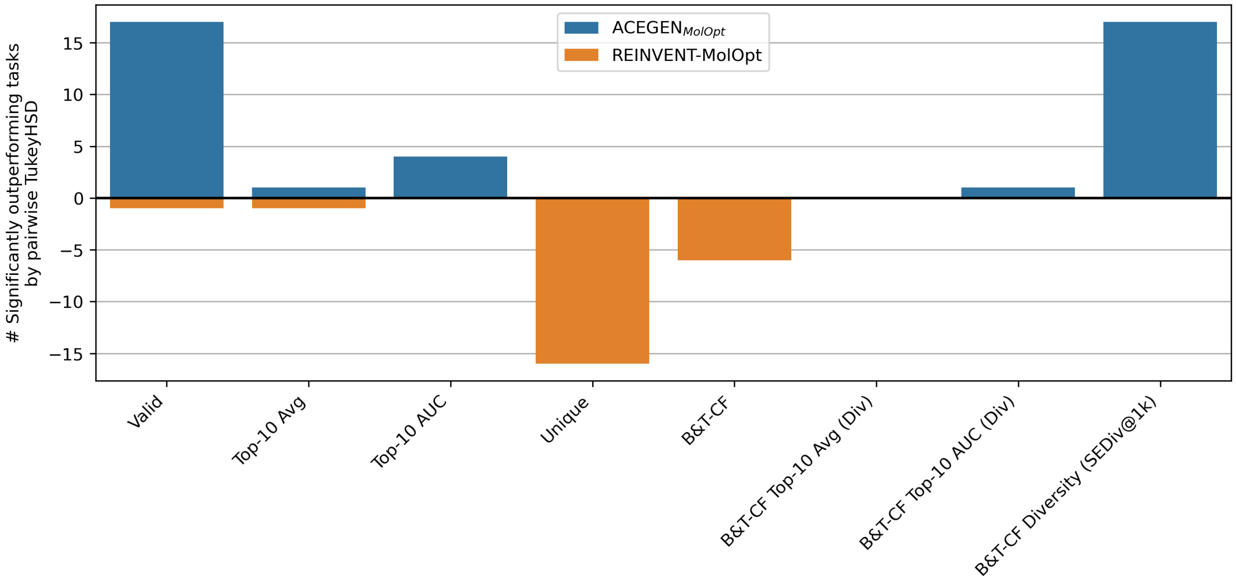

Figure S5: Per task significance performance comparison between ACEGEN$_{MolOpt}$ and REINVENT-MolOpt by pairwise TukeyHSD on key metrics. Positive count means ACEGEN$_{MolOpt}$ peformed better, negative count means REINVENT-MolOpt performed better. For example, ACEGEN$_{MolOpt}$ performs significantly better than REINVENT-MolOpt in Top-10 AUC in 4 out of 23 tasks. Note that although REINVENT-MolOpt generates significantly more unique molecules in 16 tasks, ACEGEN$_{MolOpt}$ generates significantly more diverse solutions in 16 tasks.

E Optimizing Boltz2 estimated binding affinity

E.1 Boltz2 reward function

To use Boltz2[S1] as a reward function, we integrated Boltz2 into MolScore[S2] adding it to the suite of other scoring functions available. The yaml files to run Boltz2 co-folding and estimated binding affinity for ATP-competitive and allosteric can be found in Listing 1 and 2, respectively. The reward for molecules was calculated as the arithmetic mean of several properties (p_i) the estimated binding affinity, the estimated binding probability, and the QED[S3] (in order to encourage molecules to maintain drug-like properties). The QED and and estimated binding probability are already in the range [0, 1], however, estimated affinity is in the range [2, -3]. In this case, estimated affinity values correspond to the $\log_{10}$ scale of potency centered at 1 μM. Hence 2 has a potency of 100 μM and -3 has a potency of 1 nM. This is therefore transformed to a range [0,1] by maxmin normalization, where 1 is the best (lowest) value observed, and 0 is the worst (highest) value observed. The maximum and minimum values are updated throughout learning as new molecules are sampled. Lastly, to encourage diverse candidate solutions, we use a ScaffoldSimilarityECFP diversity filter (described elsewhere[S2,S4]) to penalize the reward when already commonly observed molecular scaffolds and resampled. The full hyperparameters for this reward mechanism are available in Listing 3 which can be passed to MolScore to exactly reproduce the drug design task (alongside the Boltz2 YAML files).

$$R(x) = \frac{1}{n}\sum_{i=1}^{n} p_i(x) \tag{1}$$

```yaml
version: 1  # Optional, defaults to 1
sequences:
  - protein:
      id: [A]
      sequence: LHFLYYCSEPTLDVKIAFCQGFDKQVDVSYIAKHYNMSKSKVDNQFYSVEVGDSTFTVLKRYQNLKPIGSGAQGIVCAAYDAVLDRNVAIK
      ↪ KLSRPFQNQTHAKRAYRELVLMKCVNHKNIISLLNVFTPQKTLEEFQDVYLVMELMDANLCQVIQMELDHERMSYLLYQMLCGIKHLHSAGIIHRDL
      ↪ KPSNIVVKSDCTLKILDFGLARTAGTSFMMTPYVVTRYYRAPEVILGMGYKENVDIWSVGCIMGEMVRHKILFPGRDYIDQWNKVIEQLGTPCPEFM
      ↪ KKLQPTVRNYVENRPKYAGLTFPKLFPDSLFPADSEHNKLKASQARDLLSKMLVIDPAKRISVDDALQHPYINVWYDPAEVEAPPPQIYDKQLDERE
      ↪ HTIEEWKELIYKEVMNSEEKTKNGVVKGQPSPSGAAVNSSESLPPSSSVNDISSMSTDQTLASDTDSSLEASAGPLGCCR
properties:
  - affinity:
    binder: Z
```

Listing 1: Boltz2 YAML configuration for ATP-competitive JNK3 co-folding.

```yaml
version: 1  # Optional, defaults to 1
sequences:
  - protein:
      id: [A]
      sequence: LHFLYYCSEPTLDVKIAFCQGFDKQVDVSYIAKHYNMSKSKVDNQFYSVEVGDSTFTVLKRYQNLKPIGSGAQGIVCAAYDAVLDRNVAIK
      ↪ KLSRPFQNQTHAKRAYRELVLMKCVNHKNIISLLNVFTPQKTLEEFQDVYLVMELMDANLCQVIQMELDHERMSYLLYQMLCGIKHLHSAGIIHRDL
      ↪ KPSNIVVKSDCTLKILDFGLARTAGTSFMMTPYVVTRYYRAPEVILGMGYKENVDIWSVGCIMGEMVRHKILFPGRDYIDQWNKVIEQLGTPCPEFM
      ↪ KKLQPTVRNYVENRPKYAGLTFPKLFPDSLFPADSEHNKLKASQARDLLSKMLVIDPAKRISVDDALQHPYINVWYDPAEVEAPPPQIYDKQLDERE
      ↪ HTIEEWKELIYKEVMNSEEKTKNGVVKGQPSPSGAAVNSSESLPPSSSVNDISSMSTDQTLASDTDSSLEASAGPLGCCR
  - ligand:
      id: [B]
      smiles:  Nc1ncnc2c1ncn2[C@@H]1O[C@H](COP(=O)(O)OP(=O)(O)OP(=O)(O)O)[C@@H](O)[C@H]1O # ATP
properties:
  - affinity:
    binder: Z
```

Listing 2: Boltz2 YAML configuration for allosteric JNK3 co-folding with ATP stated to bind to the ATP site.

```
{
  "task": "JNK3_binding",
  "output_dir": "./",
  "load_from_previous": false,
  "logging": false,
  "monitor_app": false,
  "termination_exit": false,
  "scoring_functions": [
    {
      "name": "Boltz",
      "run": true,
      "parameters": {
        "input_path": "/path/to/boltz_affinity.yaml",
        "prefix": "Boltz",
        "env_engine": "mamba",
        "cache": "~/.boltz",
        "devices": [
          "0",
          "1",
          "2",
          "3"
        ],
        "accelerator": "gpu",
        "recycling_steps": 3,
        "sampling_steps": 200,
        "diffusion_samples": 1,
        "step_scale": 1.638,
        "output_format": "mmcif",
        "num_workers": 2,
        "override": false,
        "use_msa_server": true,
        "msa_server_url": "https://api.colabfold.com",
        "msa_pairing_strategy": "greedy",
        "write_full_pae": false,
        "write_full_pde": false
      }
    },
    {
      "name": "MolecularDescriptors",
      "run": true,
```

```
    "parameters": {
      "prefix": "desc",
      "n_jobs": 1
    }
  }
],
"scoring": {
  "metrics": [
    {
      "name": "Boltz_affinity_pred_value",
      "filter": false,
      "weight": 1.0,
      "modifier": "norm",
      "parameters": {
        "objective": "minimize"
      }
    },
    {
      "name": "Boltz_affinity_probability_binary",
      "filter": false,
      "weight": 1.0,
      "modifier": "raw",
      "parameters": {}
    },
    {
      "name": "desc_QED",
              "filter": false,
      "weight": 1.0,
      "modifier": "raw",
      "parameters": {}
    }
  ],
  "method": "amean"
},
"diversity_filter": {
  "run": true,
  "name": "ScaffoldSimilarityECFP",
  "parameters": {
    "nbmax": 50,
    "minscore": 0.5,
```

```
      "minsimilarity": 0.8,
      "radius": 2,
      "useFeatures": false,
      "bits": 1024,
      "outputmode": "linear"
    }
  }
}
```

Listing 3: MolScore configuration file for the JNK3 binding affinity task. This can be used to reproduce the same scoring/evaluation mechanism.

E.2 SynFlowNet

We re-implemented SynFlowNet[S5] with code forked from `https://github.com/mirunacrt/synflownet`. Minimal changes were made to the code to use MolScore[S2] to calculate the reward for SMILES, ensuring equal objective design across algorithms. Note that MolScore returns a score in the range [0,1] whilst SynFlowNet expected a score in the range [0, 100], therefore scores were transformed to match the expected range in SynFlowNet, constituting the only notable code change. None of the default parameters were modified which are listed in Listing 4. To ensure that SynFlowNet was working as expected, we ran additional experiments on two tasks reported in the original publication, GSK3β and DRD2. Table S15 shows similar performance as expected, in one case slightly worse, in the other case slightly better. In our case we ran GSK3β and DRD2 as implemented in MolScore which may lead to small deviations, however, most importantly similar learning is observed within a budget of 10,000 molecule evaluations.

```
desc: noDesc
log_dir: ./logs/debug_run_reactions_task_2025-07-08_00-20-43
resume: null
device: cuda
seed: 43
validate_every: 5000
```

```yaml
checkpoint_every: null
store_all_checkpoints: false
print_every: 1
start_at_step: 0
num_final_gen_steps: 0
num_validation_gen_steps: 10
num_training_steps: 156
num_workers: 0
hostname: localhost
pickle_mp_messages: false
mp_buffer_size: 536870912
git_hash: 3df8f52
overwrite_existing_exp: true
algo:
  method: TB
  num_from_policy: 64
  num_from_dataset: 0
  num_from_buffer_for_pb: 64
  valid_num_from_policy: 64
  valid_num_from_dataset: 0
  max_len: 3
  max_nodes: 9
  max_edges: 128
  illegal_action_logreward: -75.0
  illegal_bck_traj_reward: -1.0
  bck_reward_exponent: 1.0
  synthesis_cost_as_bck_reward: false
  strict_forward_policy: false
  strict_bck_masking: false
  train_random_action_prob: 0.0
  train_det_after: null
  valid_random_action_prob: 0.0
  sampling_tau: 0.99
  tb:
    bootstrap_own_reward: false
    epsilon: null
    reward_loss_multiplier: 1.0
    variant: TB
    do_correct_idempotent: false
    do_parameterize_p_b: true
```

```yaml
    do_predict_n: false
    do_sample_p_b: false
    do_length_normalize: false
    subtb_max_len: 128
    Z_learning_rate: 0.001
    Z_lr_decay: 50000.0
    cum_subtb: true
    loss_fn: MSE
    loss_fn_par: 1.0
    n_loss: none
    n_loss_multiplier: 1.0
    reinforce_loss_multiplier: 1.0
    bck_entropy_loss_multiplier: 1.0
    mle_loss_multiplier: 1.0
    backward_policy: MaxLikelihood
model:
  num_layers: 4
  num_emb: 128
  dropout: 0.0
  graph_transformer:
    num_heads: 2
    ln_type: pre
    num_mlp_layers: 0
    concat_heads: true
    continuous_action_embs: true
    fingerprint_type: morgan_1024
    fingerprint_path: null
opt:
  opt: adam
  learning_rate: 0.0001
  lr_decay: 2000.0
  weight_decay: 1.0e-08
  momentum: 0.9
  clip_grad_type: norm
  clip_grad_param: 10.0
  adam_eps: 1.0e-08
replay:
  use: false
  capacity: 10000
  warmup: 64
```

```yaml
  hindsight_ratio: 0.0
  num_from_replay: 0
  num_new_samples: 64
task:
  reactions_task:
    templates_filename: hb.txt
    reverse_templates_filename: null
    reward: null # Calculated by MolScore
    building_blocks_filename: enamine_bbs.txt
    precomputed_bb_masks_filename: precomputed_bb_masks_enamine_bbs.pkl
    building_blocks_costs: null
    sanitize_building_blocks: false
cond:
  valid_sample_cond_info: true
  temperature:
    sample_dist: constant
    dist_params:
    - 32.0
    num_thermometer_dim: 32
  moo:
    num_objectives: 2
    num_thermometer_dim: 16
  weighted_prefs:
    preference_type: dirichlet
    preference_param: 1.5
  focus_region:
    focus_type: centered
    use_steer_thermomether: false
    focus_cosim: 0.98
    focus_limit_coef: 0.1
    focus_model_training_limits:
    - 0.25
    - 0.75
    focus_model_state_space_res: 30
    max_train_it: 20000
reward: null # Calculated by MolScore
```

Listing 4: Default hyperparameters used for SynFlowNet. Note that the reward parameter is ignored as this is calculated using MolScore instead.

Table S15: Using SynFlowNet in this work on two example tasks reported in the original publication[S5]. In our case, GSK3β performs slightly worse, but DRD2 performs slightly better. Likewise we ran the tasks in 3 replicates.

Task	SynFlowNet[S5] (reported)	SynFlowNet (our findings)
GSK3β	0.691 ± 0.034	0.567 ± 0.041
DRD2β	0.885 ± 0.027	0.927 ± 0.018

E.3 De novo generated compounds

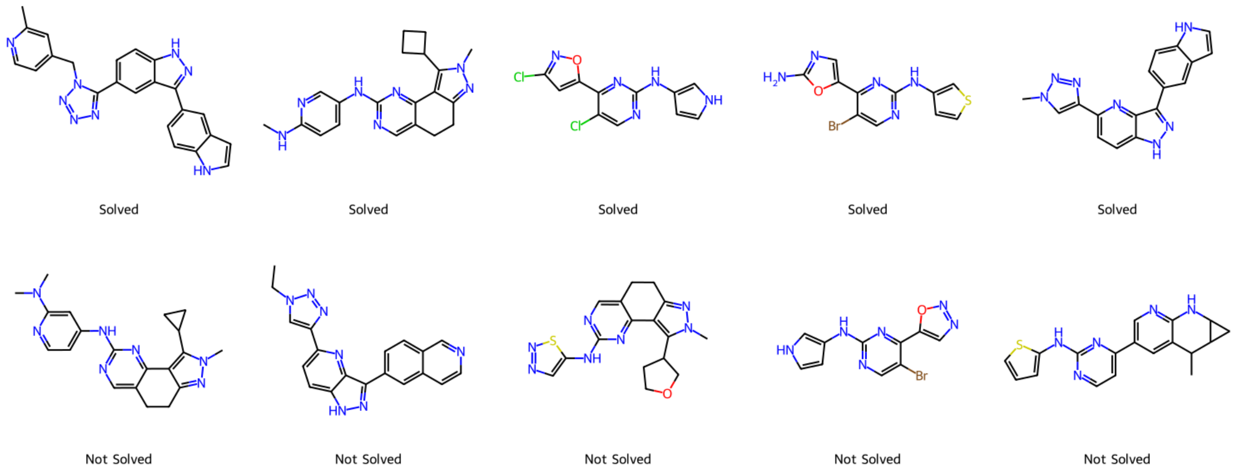

Figure S6: Examples of AiZynthFinder solved (top row) and unsolved (bottom row) ACEGEN-generated compounds from the top 100 predicted orthosteric binders (ATP-site).

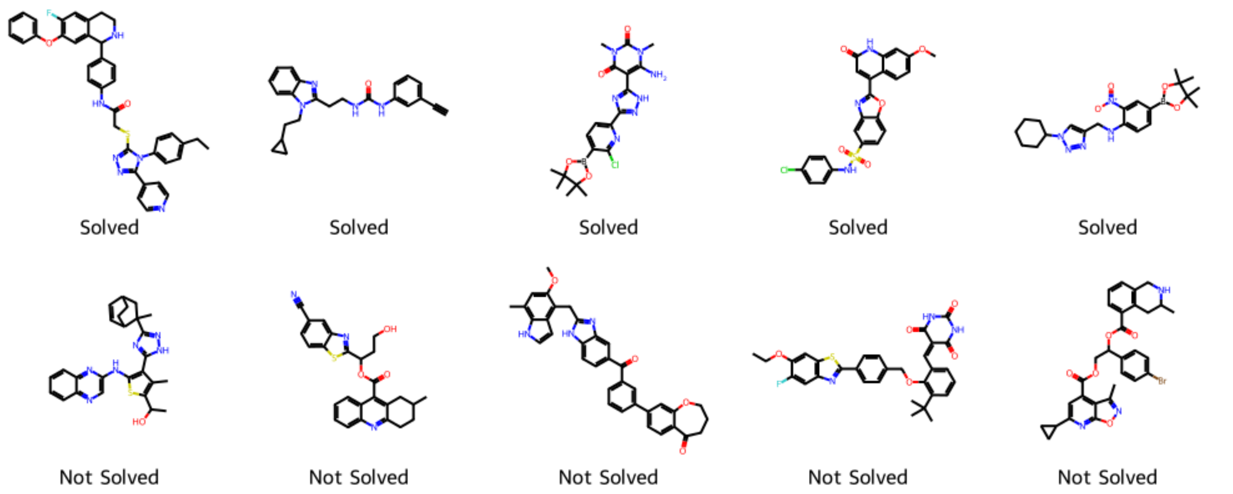

Figure S7: Examples of AiZynthFinder solved (top row) and unsolved (bottom row) SynFlowNet-generated compounds from the top 100 predicted orthosteric binders (ATP-site).

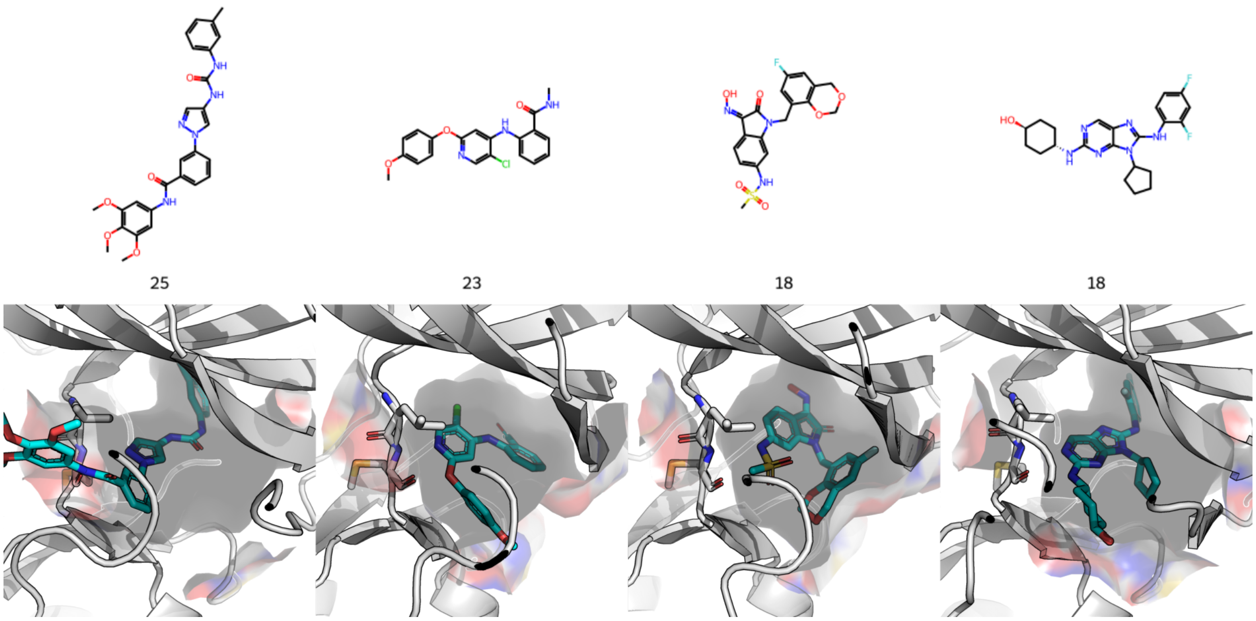

Figure S8: Examples of JNK3 ligands and their co-folded structure into the orthosteric pocket (ATP-site). Centroids of the 4 largest clusters are shown, alongside the number of cluster members. Molecules were clustered via Butina clustering of the ECFP4 fingerprints of their Bemis-Murcko scaffolds (using a distance threshold of 0.25)

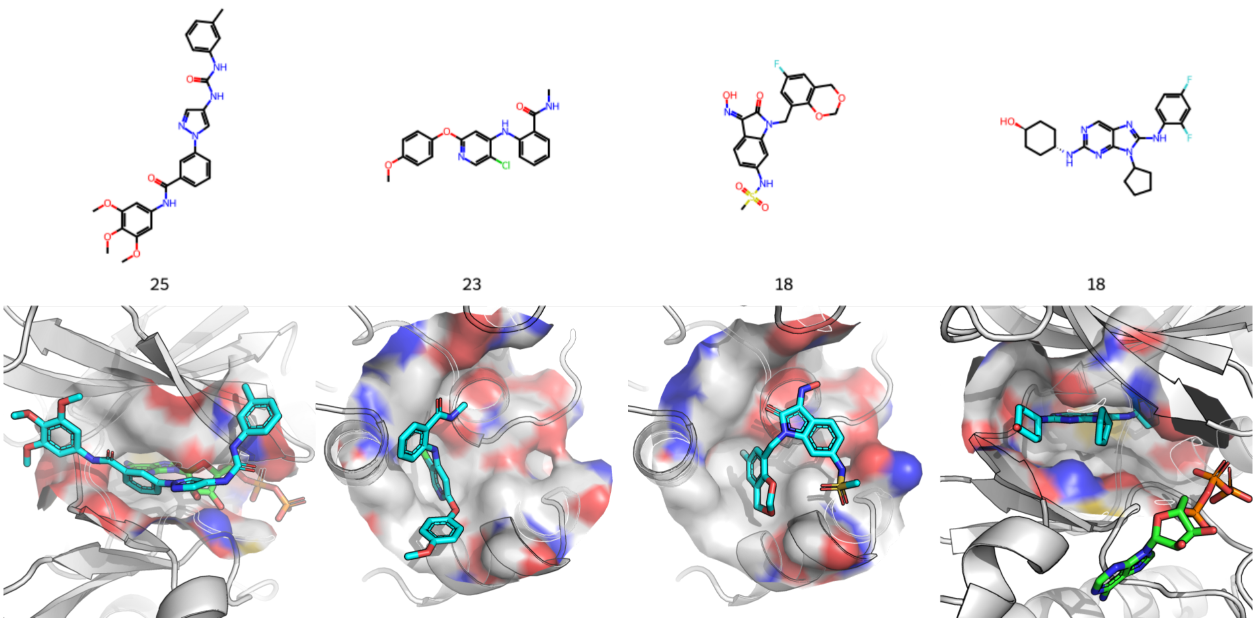

Figure S9: Examples of JNK3 ligands and their co-folded structure into an allosteric pocket (i.e., co-folded with ATP). Centroids of the 4 largest clusters are shown, alongside the number of cluster members. Molecules were clustered via Butina clustering of the ECFP4 fingerprints of their Bemis-Murcko scaffolds (using a distance threshold of 0.25)

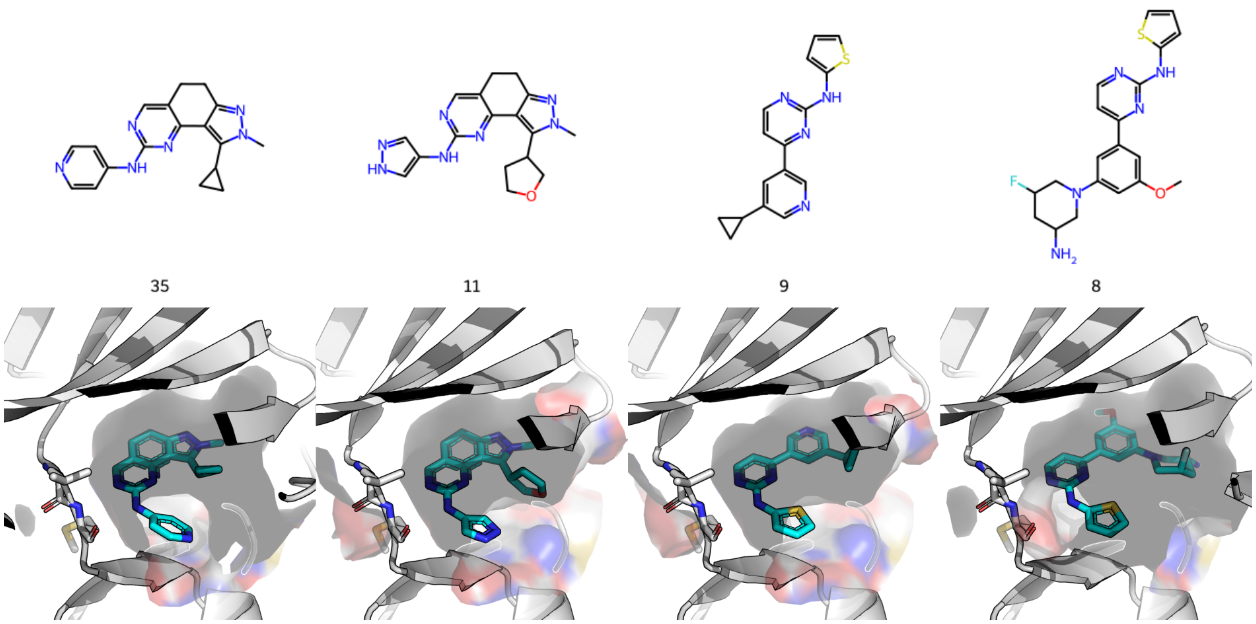

Figure S10: Examples of de novo compounds and their co-folded structure into the orthosteric pocket (ATP-site). Centroids of the 4 largest clusters are shown, alongside the number of cluster members. Molecules were clustered via Butina clustering of the ECFP4 fingerprints of their Bemis-Murcko scaffolds (using a distance threshold of 0.25)

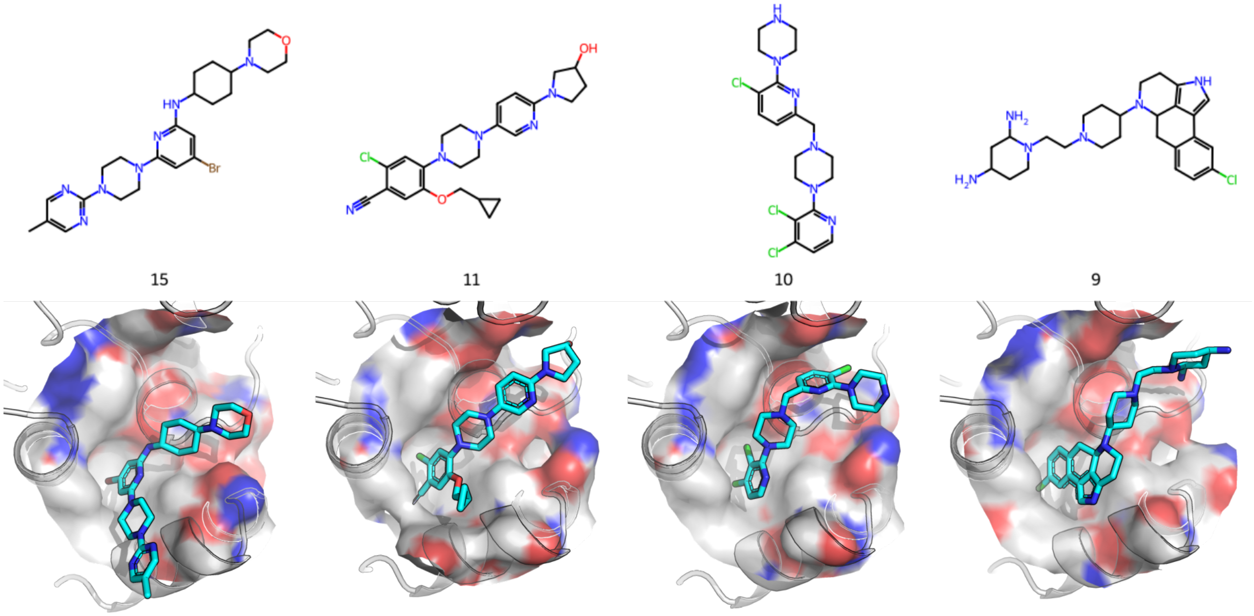

Figure S11: Examples of de novo compounds and their co-folded structure into an allosteric pocket (i.e., co-folded with ATP). Centroids of the 4 largest clusters are shown, alongside the number of cluster members. Molecules were clustered via Butina clustering of the ECFP4 fingerprints of their Bemis-Murcko scaffolds (using a distance threshold of 0.25)

E.4 Absolute binding free energy

Preparation of the co-folded structures was done using Schrödinger protein preparation wizard[S6]. This preparation step involves assigning bond orders, adding hydrogens, creating disulfide bonds, generating most likely tautomer and protonation state, and a restrained minimization using OPLS4 forcefield. AFEP calculations were done using FEP+ suite from Schrödinger[S7] with default parameters; chemical potential ensemble, 5 ns lambda windows, and 1 ns replica exchange solute tempering molecular dynamics for enhanced sampling. Both steps were performed using 2024-4 Schrödinger release.

A random subset of up to 10 ACEGEN generated JNK3 putative orthosteric and allosteric compounds was selected within a range of ± 0.25 from four Boltz2 estimated affinity values (5, 6, 7, 8). To ensure similar physicochemical properties, molecules were sampled from a heavy atom count range between 25 and 35, and a logP range between 2 and 4 (calculated using cLogP[S8]). Compounds that failed ABFE simulations were removed. Remaining compounds selected are shown in Figure S12 - Figure S19.

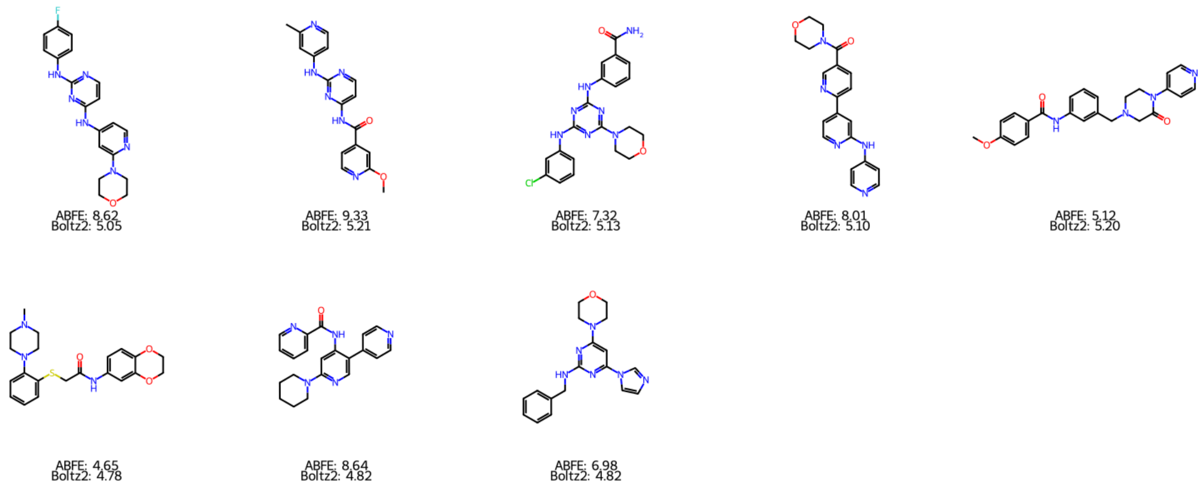

Figure S12: Selected orthosteric compounds for validation with AFEP at a Boltz2 affinity of 5 ± 0.25.

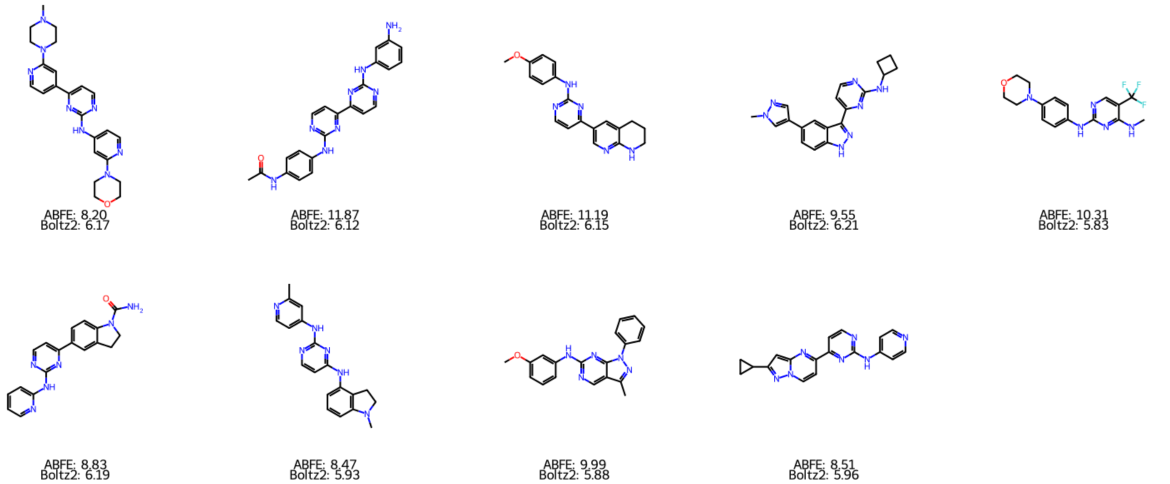

Figure S13: Selected orthosteric compounds for validation with AFEP at a Boltz2 affinity of 6 ± 0.25.

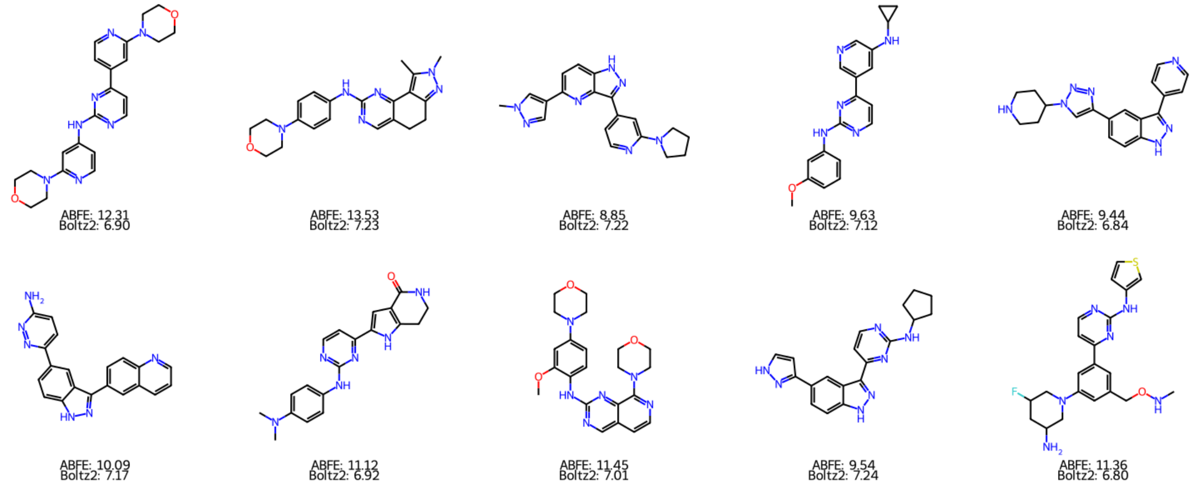

Figure S14: Selected orthosteric compounds for validation with AFEP at a Boltz2 affinity of 7 ± 0.25.

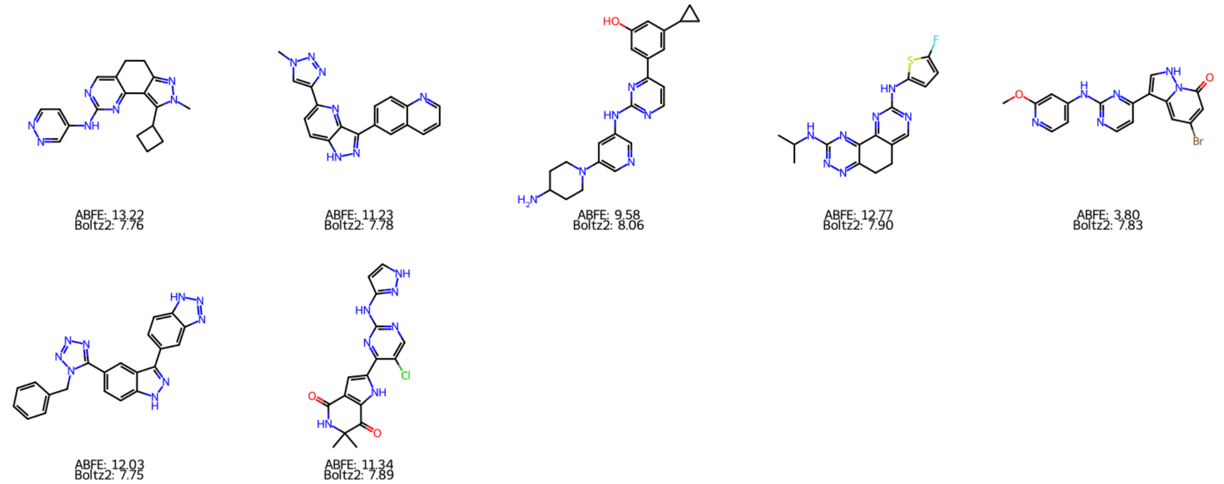

Figure S15: Selected orthosteric compounds for validation with AFEP at a Boltz2 affinity of 8 ± 0.25.

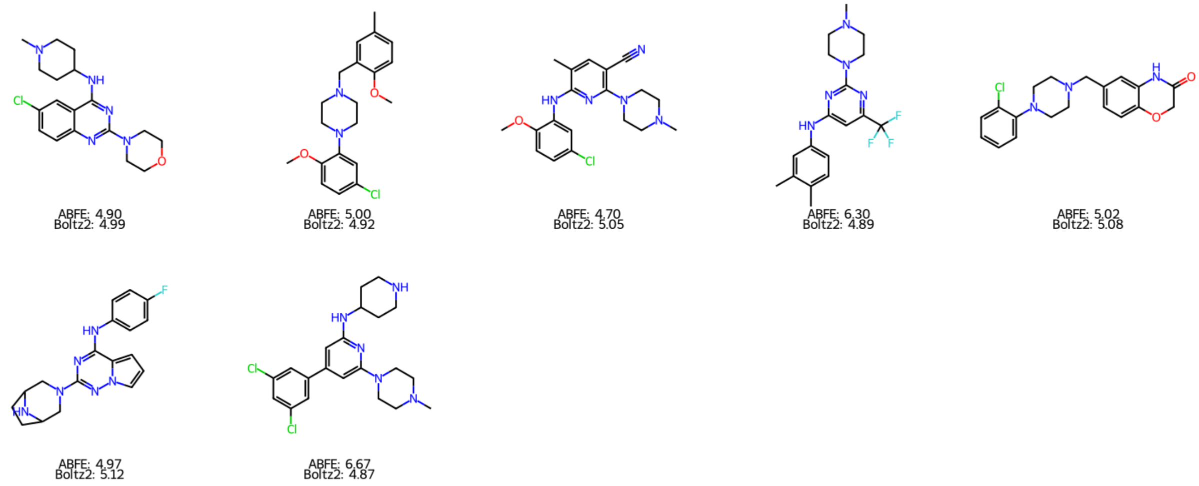

Figure S16: Selected allosteric compounds for validation with AFEP at a Boltz2 affinity of 5 ± 0.25.

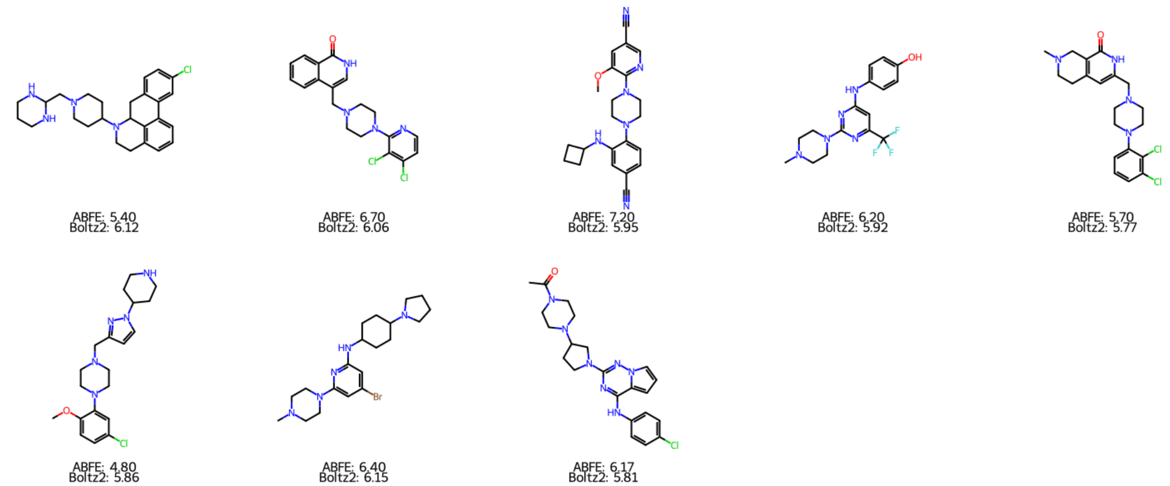

Figure S17: Selected allosteric compounds for validation with AFEP at a Boltz2 affinity of 6 ± 0.25.

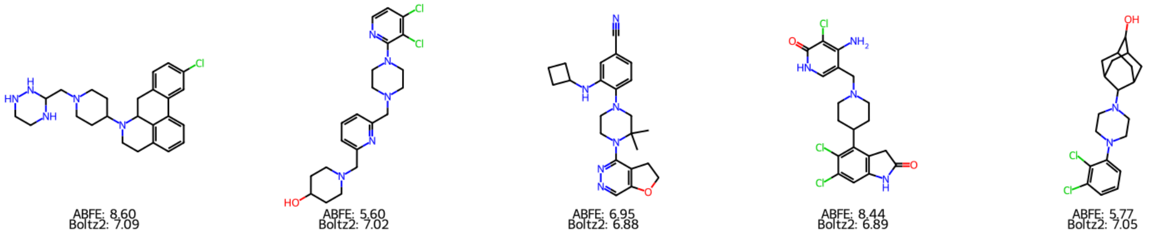

Figure S18: Selected allosteric compounds for validation with AFEP at a Boltz2 affinity of 7 ± 0.25.

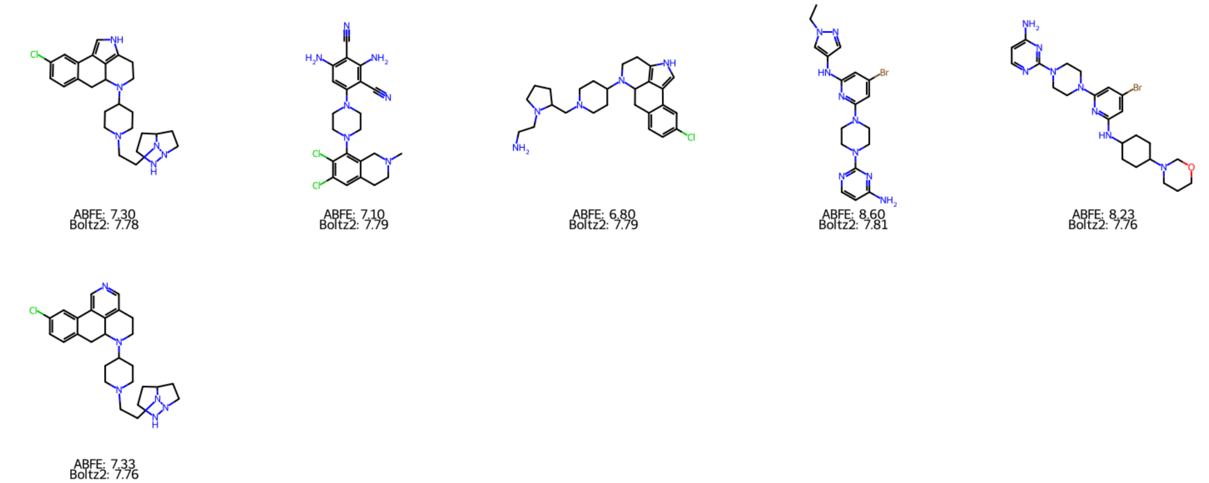

Figure S19: Selected allosteric compounds for validation with AFEP at a Boltz2 affinity of 8 ± 0.25.

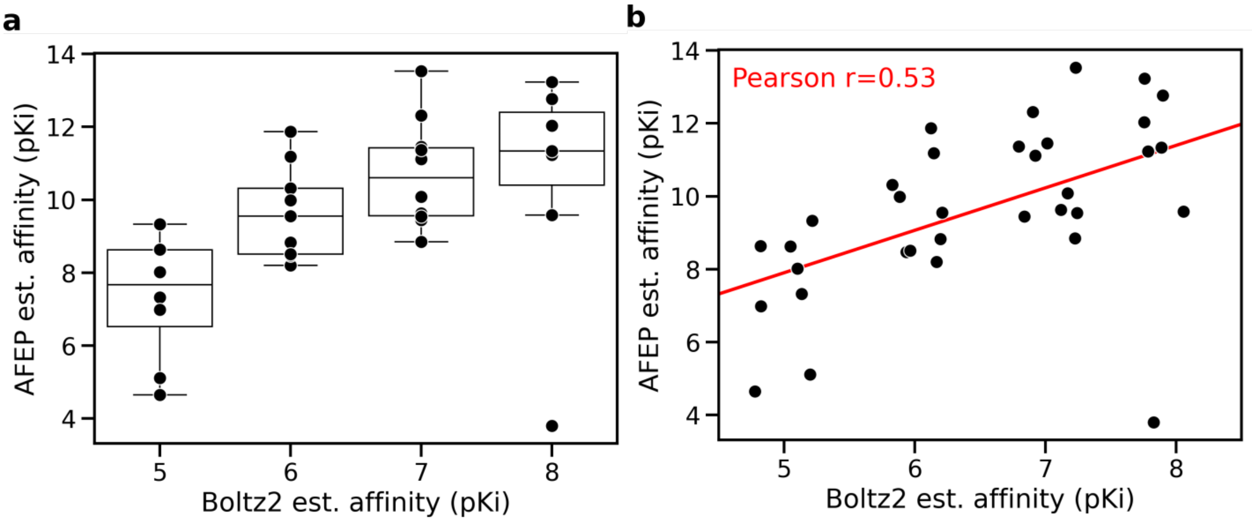

Figure S20: Correlation between Boltz2 estimated pKi and ABFE estimated pKi. (a) Binned affinities show a positive stepwise correlation, where a one-way ANOVA test reports significant variance between the means (p=0.0049). (b) The non-binned correlation between Boltz2 estimated affinity and AFEP with a reported Pearson correlation of 0.53.

References

(S1) Passaro, S.; Corso, G.; Wohlwend, J.; Reveiz, M.; Thaler, S.; Ram Somnath, V.; Getz, N.; Portnoi, T.; Roy, J.; Stark, H.; others Boltz-2: Towards Accurate and Efficient Binding Affinity Prediction. *BioRxiv* **2025**, 2025–06.

(S2) Thomas, M.; O'Boyle, N. M.; Bender, A.; De Graaf, C. MolScore: A scoring and evaluation framework for de novo drug design. **2023**,

(S3) Bickerton, G. R.; Paolini, G. V.; Besnard, J.; Muresan, S.; Hopkins, A. L. Quantifying the chemical beauty of drugs. *Nature chemistry* **2012**, *4*, 90–98.

(S4) Thomas, M.; O'Boyle, N. M.; Bender, A.; De Graaf, C. Augmented Hill-Climb increases reinforcement learning efficiency for language-based de novo molecule generation. *Journal of Cheminformatics* **2022**, *14*, 1–22.

(S5) Cretu, M.; Harris, C.; Igashov, I.; Schneuing, A.; Segler, M.; Correia, B.; Roy, J.; Bengio, E.; Liò, P. Synflownet: Design of diverse and novel molecules with synthesis constraints. *arXiv preprint arXiv:2405.01155* **2024**,

(S6) Madhavi Sastry, G.; Adzhigirey, M.; Day, T.; Annabhimoju, R.; Sherman, W. Protein and ligand preparation: parameters, protocols, and influence on virtual screening enrichments. *Journal of computer-aided molecular design* **2013**, *27*, 221–234.

(S7) Chen, W.; Cui, D.; Jerome, S. V.; Michino, M.; Lenselink, E. B.; Huggins, D. J.; Beautrait, A.; Vendome, J.; Abel, R.; Friesner, R. A.; others Enhancing hit discovery in virtual screening through absolute protein–ligand binding free-energy calculations. *Journal of Chemical Information and Modeling* **2023**, *63*, 3171–3185.

(S8) Wildman, S. A.; Crippen, G. M. Prediction of physicochemical parameters by atomic contributions. *Journal of chemical information and computer sciences* **1999**, *39*, 868–873.
